# Patterned Arrangements of Olfactory Receptor Gene Expression in Zebrafish are Established by Radial Movement of Specified Olfactory Sensory Neurons

**DOI:** 10.1038/s41598-017-06041-1

**Published:** 2017-07-17

**Authors:** Xalid Bayramli, Yiğit Kocagöz, Uğurcan Sakizli, Stefan H. Fuss

**Affiliations:** 1 Bogaziçi University, Department of Molecular Biology and Genetics, 34342 Bebek, Istanbul, Turkey; 2 Center for Life Sciences and Technologies, 34342 Bebek, Istanbul, Turkey

## Abstract

Spatial restriction of olfactory receptor (OR) gene expression in peripheral sense organs is a common phenomenon across species, suggesting that zonal OR expression somehow contributes to olfactory function. In zebrafish OR expression patterns reminiscent of zones occur as concentric domains with preferred diameters for different ORs. However, the function and the developmental origin of the pattern are unknown. Here we investigate olfactory sensory neuron (OSN) neurogenesis in the adult zebrafish olfactory epithelium (OE) to understand how the zonally organized OR pattern is established and maintained during the lifetime of the animal. We find that OSNs are generated from two discontinuous proliferation zones located at the central and peripheral edge of the sensory OE. OSNs turn on OR expression soon after they exit mitosis and invade the sensory tissue, approaching each other from both ends of the OE. Biased generation of OSN subpopulations at both neurogenic sites and elimination of OSNs along their route across the OE generates the impression of OR-specific expression domains. We formulated a simple mathematical model based on exact parameters derived from our analysis of OSN neurogenesis, which accurately generates OR-like distributions without the need to invoke molecular signals to pattern the OE.

## Introduction

The functional architecture of the olfactory system is remarkably similar across phyla as disparate as insects, amphibians, fish, and mammals, suggesting that common organizing principles serve crucial aspects of olfactory function. Typically, olfactory sensory neurons (OSNs) express only one or a specific combination of a few chemoreceptors from a large and diverse repertoire^1–3^ and axons of OSNs expressing the same olfactory receptor (OR) converge onto the same glomerulus in the vertebrate olfactory bulb^4^ or insect antennal lobe^5, 6^. These fundamental principles are believed to sharpen sensory acuity at the level of individual OSNs, to increase the sensitivity to odorants at the level of glomeruli, to enhance discrimination of odorants by lateral processing in the olfactory bulb, and to provide a spatially organized input pattern for higher level processing of olfactory information.

A less well understood, but equally common, phenomenon is the spatial organization of OR gene expression patterns in peripheral sense organs. Typically, OSNs expressing the same OR are not evenly scattered across the surface of the olfactory epithelium (OE) but are restricted to confined domains, commonly referred to as ‘zones’. Patterns of segregated OR expression have been described for the *Drosophila* antenna^6^ and the OE of frogs^7^, salamanders^8^, rodents^9, 10^ and zebrafish^11^. A comprehensive analysis in the mouse OE revealed distinct but partially overlapping expression patterns organized as a continuum along the dorso-medial to ventro-lateral axis for nearly each of the 80 ORs examined^12^. A correlate of zonal OR expression is found in zebrafish in which OSNs expressing different ORs occupy preferred concentric domains with OR-specific diameters^11^.

The functional significance of spatially restricted OR expression remains elusive but physiological and developmental roles have been proposed. According to the sorption hypothesis, ORs are positioned in the OE with respect to their ligand profiles and the likelihood with which a ligand could interact best with a receptor based on local airflow and physicochemical properties of the ligand^13^. Patterned OR expression in the OE may also be crucial for proper formation of glomeruli in the olfactory bulb and a zone-to-zone correlation between the OE and positions of glomeruli along the dorso-ventral axis of the olfactory bulb has been demonstrated^12, 14^. Alternatively, zonal OR expression may reflect a less-well understood step in the hierarchy of molecular mechanism that ensure monogenic OR expression by limiting the number of OR genes from which a given OSN can choose based on its position in the OE.

The robustness of defined OR expression domains is remarkable in the light that the OE undergoes constant turnover due to the limited and short life span of OSNs of approximately 30 to 90 days^15, 16^. Therefore, processes or molecular signals that pattern the embryonic OE should be maintained during postnatal life. In the rodent OE new OSNs are generated from basally located stem/progenitor cell populations^17^ and ascend to more apical positions as they adopt functional maturity^18^. Because their position does not shift laterally, OR-specific OSN subpopulations must be generated locally, either from lineage-restricted stem/progenitor cells or through yet unidentified determination factors that influence OR gene choice in a spatially defined manner. In zebrafish, regions of high proliferative activity have been described at the edge of the sensory OE^19–21^, thus, in positions that do not overlap with OR expression. The contribution of cells generated at these sites to the neuronal population, however, has not been investigated.

Here we examine OSN neurogenesis in the post larval zebrafish OE quantitatively and with high spatial resolution to understand the generation of OR-specific, spatially restricted, ring-shaped expression domains. We show that OSNs are generated from two discontinuous neurogenic sites, located at the central and peripheral edge of the sensory OE. OSNs turn on chemoreceptor expression shortly after they exit the cell cycle and subsequently invade the sensory OE to establish specific distribution patterns that are reminiscent of zones. We propose a simple mathematical model to describe the segregation of OSN populations over time, which is governed largely by spatial bias in OSN neurogenesis, the dynamics of positional shifts of newborn OSNs, and OSN life span. We conclude that the zone-like OR expression pattern in the zebrafish OE is the outcome of simple developmental processes and tissue dynamics but does not depend on morphogenetic molecular patterns laid out in the tissue.

## Results

### Proliferative activity in the adult zebrafish OE

**Figure 1:**
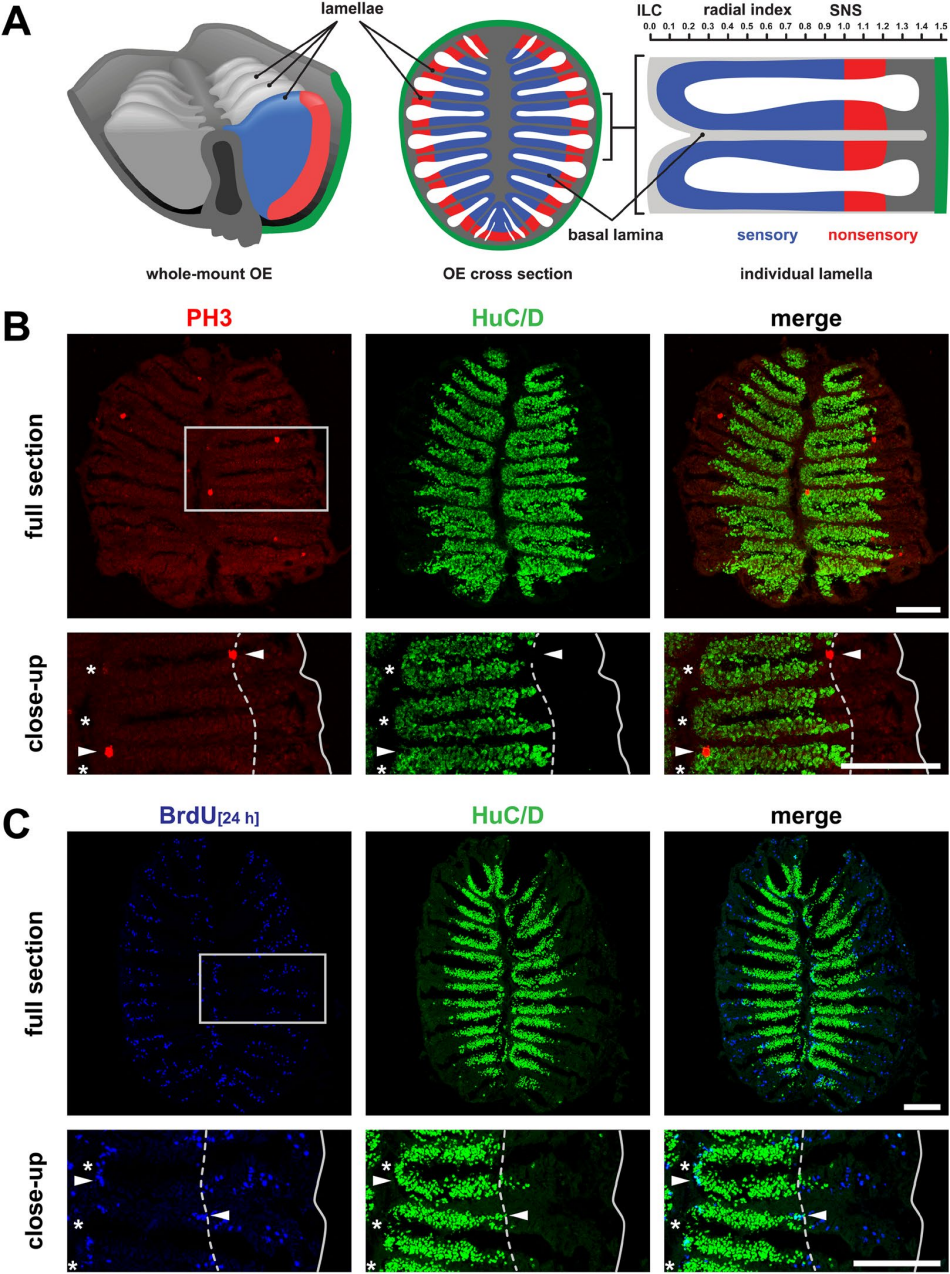
Cell proliferation in the adult zebrafish olfactory epithelium. (**A**) Schematic overview of the zebrafish OE, which forms by repeated folding of the epithelial sheet to give rise to double-faced radial lamellae. The sensory epithelium of neighbouring lamellae is continuous at the interlamellar curves (ILCs) and joined at the basal lamina. Olfactory sensory neurons are restricted to the inner region (blue) sharply separated from respiratory cells in the peripheral nonsensory OE (red) at the sensory/nonsensory border (SNS). The radial index (top, right) depicts the scale for morphometric measurements of cell positions normalized to the length between the tip of ILCs and the position of the SNS. (**B**) Immunohistochemistry against the mitotic marker phospho-histone H3 (PH3, red, left) and the pan-neuronal marker HuC/D (green, centre) on full sections through the adult zebrafish OE (top) and higher power view of one side of the OE (bottom) indicated by the grey box. Mitotically active cells (arrowheads) occur at the ILCs (asterisks) and SNS (dotted line). The solid line depicts the outline of the section. Scale bars: 100 µm. (**C**) Immunohistochemistry against BrdU (blue, left) after 24 h BrdU incubation to label dividing cells and HuC/D (green, centre) to visualize mature neurons on full sections (top) and higher power views of one side of the OE (bottom) indicated by the grey box. BrdU-positive cells are restricted to the ILCs (asterisks) and SNS (dotted line) but are completely absent from the center of the sensory OE. BrdU/HuC/D double-positive cells occur at the edge of the sensory OE (arrowheads). Scale bars: 100 µm.

To identify candidate neurogenic sites in the adult zebrafish OE we stained OE cross sections by immunohistochemistry against the mitotic marker phospho-histone H3 (pH3) and the pan-neuronal marker HuC/D (Fig. 1). As reported previously^22^, mature sensory neurons reside within the central two-thirds of the tissue, while HuC/D-positive cells are completely absent from the peripheral nonsensory OE. Only few (6.6 ± 1.3 (mean ± SEM) cells/section, n = 40 sections from 4 OE of 3 fish) pH3-positive, mitotically active cells could be detected per section. Yet, pH3-positive cells were not randomly distributed across the OE but largely restricted to the central interlamellar curves (ILCs) and the periphery of the OE with a preference towards the border between the sensory and nonsensory (SNS) subregions, consistent with previous reports of high proliferative activity at these sites^20^ (Fig. 1, Supplementary Fig. 1). No HuC/pH3 double-positive cells could be observed.

**Figure 2:**
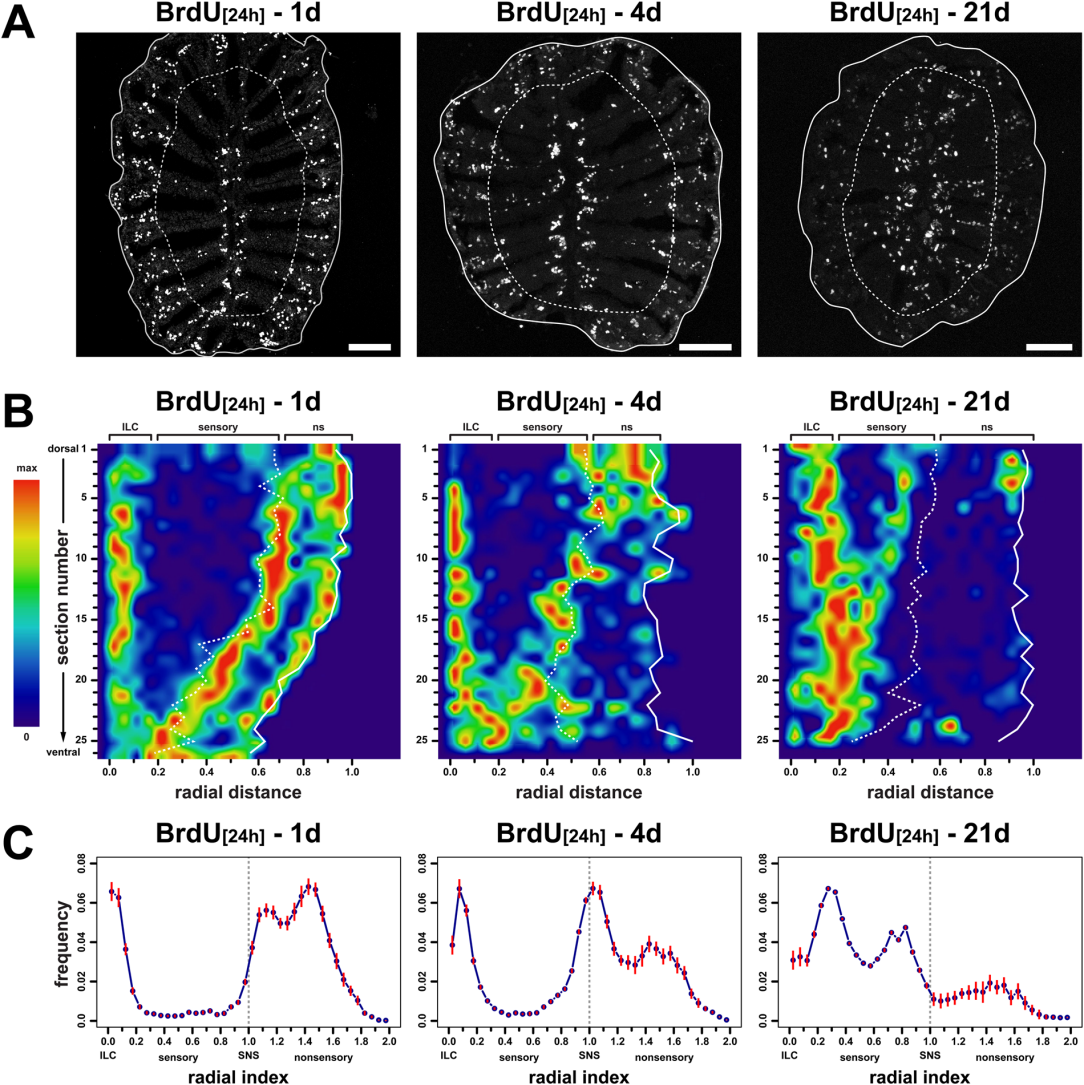
Adult-born cells invade the sensory epithelium over time. (**A**) Immunohistochemistry against BrdU following 24 h BrdU incubation 1d (left), 4d (centre), and 21d post labelling on sections through the adult OE. The dotted line indicates the position of the sensory/nonsensory border and the solid line demarcates the outline of the section. BrdU-positive cells are located at the interlamellar curves (centre of the sections) and peripherally to the sensory/nonsensory border initially but progressively invade the sensory OE. Scale bars: 100 µm. (**B**) Reconstructions of surface views onto a standardized olfactory lamella as shown in Fig. 1A 1d (left), 4d (centre), and 21d (right) following BrdU incubation for 24 h. The radial positions of BrdU-positive cells (left to right) were normalized to the maximum radius of the OE (radial distance) and plotted individually for each of 25 to 26 consecutive sections from the dorsal (top) to the ventral (bottom) margin of the OE. The circumference of the OE is indicated by a solid white line, the dotted line demarcates the average position of the SNS from all lamellae on the respective section. The frequency of BrdU-positive cells within each bin was normalized to the total number of BrdU-positive cells per section (max). The image has been smoothened with a bicubic scaling function. For a representation of unprocessed data see Supplementary Figure 2. (**C**) Frequency distributions of BrdU-positive cells 1d (left), 4d (centre), and 21d (right) following 24 h BrdU incubation. The position of labelled cells was normalized to the distance of the sensory/nonsensory border (SNS) from the tip of the interlamellar curves (ILCs). The dotted line demarcates the position of the SNS. Data points represent means ± SEM for 14.365 (1d), 10.967, (4d), and 7.463 (21d) cells from 30 sections of 3 OE for each time point. BrdU-positive cells originating from the ILC and SNS progressively invade the sensory OE over time, while cells in the periphery of the OE remain stationary. An additional population of non-migratory cells becomes visible at the ILC for the 21d time point.

PH3 immunostaining identifies acutely dividing cells but does not allow following the fate and behaviour of adult-born cells over time. Thus, cells dividing over a 24 h period were labelled by incubation with the thymidine analogue 5-Bromo-2′-deoxyuridine (BrdU), which is stably incorporated into DNA during S-phase and persists in post-mitotic cells. Consistent with the pH3 distribution pattern, BrdU-positive cells were preferentially located at the ILC and the nonsensory periphery, while the sensory OE was almost entirely devoid of labelled cells (Fig. 1). While the majority of BrdU-positive cells was HuC/D-negative, few BrdU/HuC double-positive could be detected at the ILC and SNS. When BrdU-positive cells were quantified according to their position along the radial axis of the OE, thus their distance from the central ILCs towards the periphery of the OE along individual lamellae (Fig. 1A), BrdU-positive cells showed a tri-modal distribution with a sharp peak immediately at the ILC and two distinct populations in the nonsensory OE. One of those was located outside of, but directly adjacent to the SNS, while a second population was found close to the circumference of the OE (Figs 1 and 2).

### Adult-born cells invade the sensory OE over time

Cell proliferation occurs at the central and peripheral margins of the sensory OE while mature neurons are sandwiched in between, suggesting that newborn cells invade the sensory OE over time. As a first test to understand whether cells shift positions with age, we incubated fish with BrdU for 24 h and analysed the tissue distribution of BrdU-positive cells 1d, 4d, and 21d post incubation (Fig. 2). Labelled cells are narrowly distributed at the ILC and outside the SNS immediately after BrdU incubation but disperse across the sensory OE at later time points: already 3d after the BrdU pulse, cells shifted away from the ILCs and a substantial fraction of cells crossed the SNS towards more central positions, while a punctate, seemingly random, pattern was observed three weeks after labelling (Fig. 2A).

To describe the movement of adult-born cells quantitatively, we first reconstructed representations of single olfactory lamellae (Fig. 1A) for each time point by plotting the radial and dorso-ventral positions of BrdU-positive cells from complete series of consecutive cross sections through the OE (Fig. 2B, Supplementary Fig. 2). BrdU-positive cells are arranged as tight dorso-ventral bands following the contour of the ILC, the SNS, and the circumference of the tissue immediately after BrdU incubation, but progressively and coherently shifted away from the ILC and the SNS and approached each other in the centre of the sensory OE over time.

Next, the radial positions of labelled cells from three individual fish were determined for each time point and normalized to the radial length of the sensory OE (Fig. 2C). Because the separation of proliferation zones was more pronounced in the dorsal OE (Fig. 2B) only the 10 dorsalmost sections were quantified. Immediately following BrdU incubation, on average 478.8 ± 37.2 (mean ± SEM) BrdU-positive cells (n = 30 sections from 3 OE) could be counted per section, implying that around 12.000 new cells are added to the OE each day. Three individual cell populations could be clearly recognized by the presence of distinct peaks along the distribution profiles. Two populations, initially located at the ILC and SNS, approached each other in the mid OE over time, eventually unmasking a fourth minor population that remained stationary at the ILC. Cells generated in the most peripheral OE also remained stationary and the population decreased over time.

An expectation-maximization algorithm for Gaussian mixture models was employed to estimate descriptive parameters of the underlying individual distributions (Supplementary Fig. 3). The 1d pattern was best described by a combination of three lognormal distributions with peak 1 close to the ILC at a radial index of 0.18 ± 0.31 (mean ± σ), peak 2 just outside the SNS at 1.17 ± 0.15, and peak 3 in the peripheral OE at position 1.49 ± 0.14. At the 4d time point populations 1 and 3 remained at radial positions 0.19 ± 0.20 and 1.57 ± 0.15, respectively, while peak 2 shifted towards 1.07 ± 0.18. Thus, about half of all ILC-derived cells crossed the SNS border over the short 3d period. Interestingly, at the 21d time point the ILC-derived population split into two subpopulations, one of which remained at the original ILC position of 0.17 ± 0.24 and a second population which shifted centrifugally to 0.39 ± 0.19. During the same period the SNS-derived populations advanced further to position 0.82 ± 0.14, while the peripheral population remained stationary at a radial index of 1.42 ± 0.18. The observation suggests that both proliferation sites harbour immobile stem/progenitor populations, whereas the derived differentiated cell populations undergo bidirectional shifts towards the centre of the OE.

**Figure 3:**
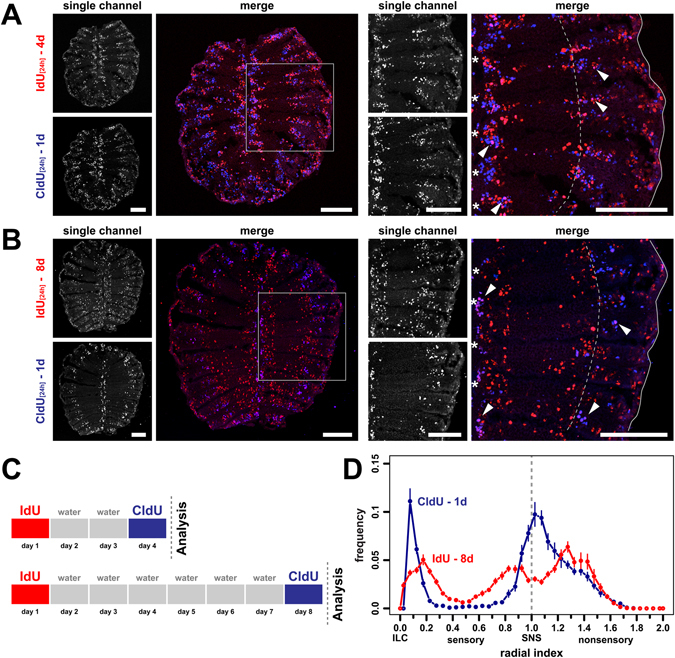
Simultaneous labeling of old and young cell populations in the same olfactory epithelium. (**A**) Cross sections through the OE of fish that were incubated with IdU (red) for 24 h followed by a second 24 h incubation with CldU (blue) two days later. Single channel exposures are shown to the left, the merged channels to the right. The panel on the right shows a higher power view of one side of the OE indicated by the grey box on the left. Single IdU- and CldU-positive cells as well as double positive cells (pink, arrowheads) can be detected. The younger CldU-positive and CldU/IdU double positive cells are confined to the interlamellar curves (asterisks) and regions peripheral to the sensory/nonsensory border (dotted line). The older single IdU-positive cells occupy more central positions in the sensory OE. Scale bars: 100 µm. (**B**) Same experiment as in A. but with a 6d time interval between consecutive labelling with IdU and CldU. IdU single-positive cells advanced further from the ILC and SNS towards the centre of the sensory epithelium. Scale bars: 100 µm. (**C**) Schematic representation of the experimental setup for A. (top) and B. (bottom). (**D**) Frequency distributions of IdU- (red) and CldU (blue) single-positive cells from the 8d experiment shown in B. The positions of cells were normalized to the length of the sensory OE between the interlamellar curves (ILCs) and the sensory/nonsensory brder (SNS). The dotted line indicates the position of the SNS. The older IdU-labeled cells show a broader, more central distribution than the younger CldU-positive cells that are concentrated at the ILC and SNS. The graph represents the positions of 4.787 IdU-positive and 3.778 CldU-positive cells from 20 sections of 2 fish, mean ± SEM.

To visualize the progressive shift in cell positions directly and in the same tissue, cells born at consecutive time points were differentially labelled by subsequent incubation with IdU and CldU interrupted by placing the fish in clean tank water for 2 or 6d (Fig. 3). For both conditions a mixture of IdU and CldU single-positive cells, which divided only once during the experiment, and IdU/CldU double-positive that divided multiple times and during both incubation periods were observed. Double-positive cells were preferentially located at the tips of the ILC or just peripheral to the SNS and, therefore, may represent stationary fast cycling stem/progenitor cells. More importantly, the older IdU single-positive cells, which most likely include terminally differentiated OSNs, located further away from the ILC and SNS and occupied more central positions than the double-positive and younger CldU single-positive cells. The distance between young and old cell populations was more pronounced when the duration between consecutive incubations with IdU and CldU was increased to 6d (Fig. 3B,D).

### Contribution of proliferation zones to neurogenesis

**Figure 4:**
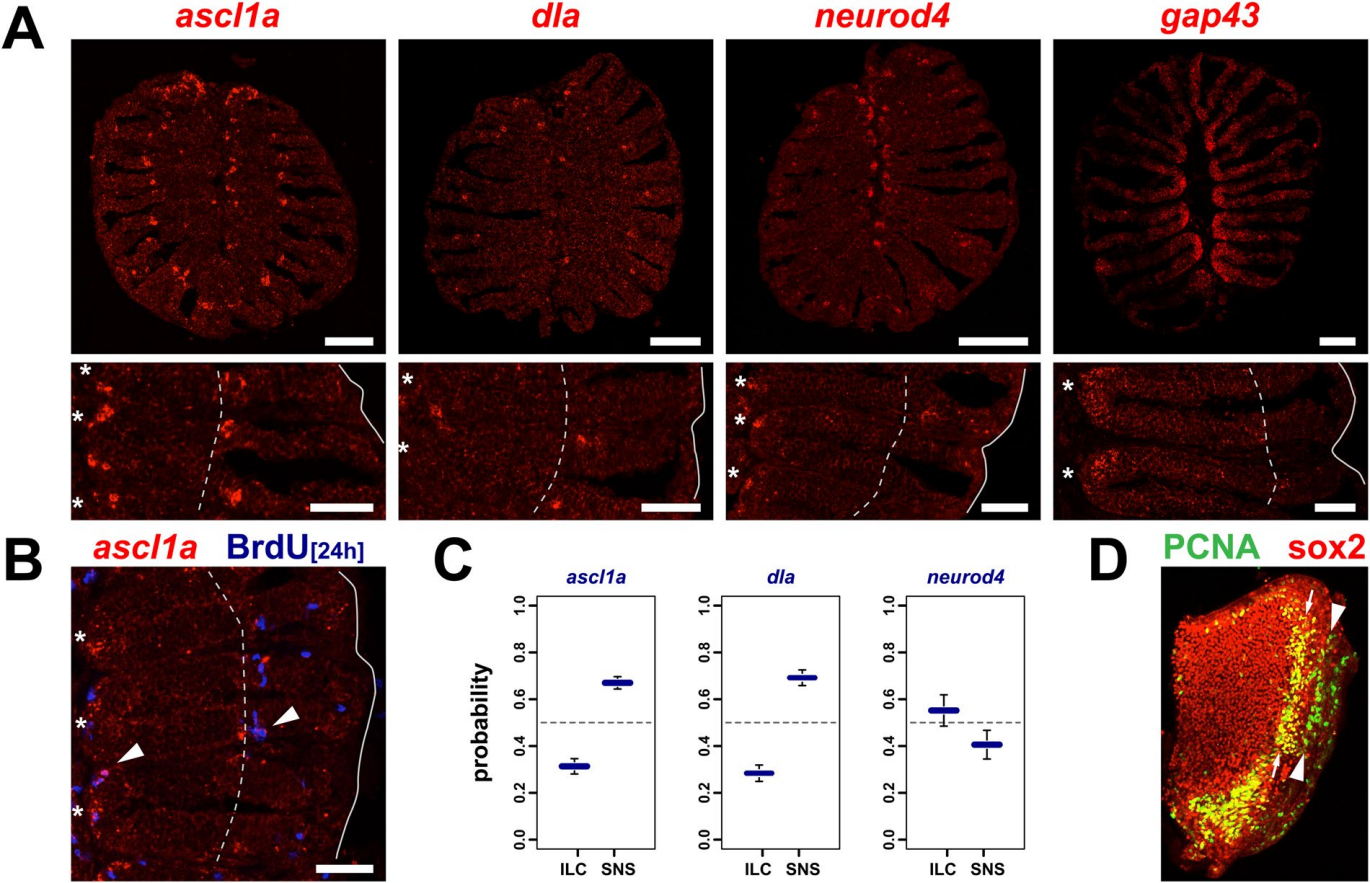
Neurogenesis is restricted to the central and peripheral edge of the sensory neuroepithelium. (**A**) *In situ*-hybridization for the early neurogenesis markers *achaete scute*-*like 1a* (*ascl1a*) and *delta*-*like a* (*dla*), and the late differentiation markers *neuronal differentiation 4* (*neurod4*) and *growth associated protein 43* (*gap43*) on full cross sections through the adult OE (top) and higher power views of one side of the OE (bottom). Expression of the neurogenic markers is restricted to the interlamellar curves (asterisks) and sensory/nonsensory border (dotted line). The solid line indicates the outline of the section. Scale bars: 100 µm (top panel), 50 µm (bottom panel). (**B**) *Ascl1a*-expressing cells are actively dividing. *In situ* hybridization for *ascl1a* (red) and immunohistochemistry against BrdU following 24 h incubation with the marker to highlight dividing cells. Double positive cells (arrowheads) can be identified at the interlamellar curves (asterisks) and the sensory/nonsensory border (dotted line). Scale bar: 50 µm. (**C**) Quantification of the distribution of cells expressing the neurogenic markers *ascl1a*, *dla*, and *neurod4* at the interlamellar curves (ILC) and sensory nonsensory border (SNS; means ± SEM for ascl1: 8 sections from 2 fish, dla: 9 sections from 3 fish, neurod4: 8 sections from 2 fish). Cells expressing the markers are unevenly distributed between the two proliferation zones; more *ascl1a*- and *dla*-expressing cells can be found at the SNS, whereas more *neurod4*-positive cells locate to the ILC. (**D**) Immunohistochemistry against the mitotic marker proliferating cell nuclear antigen (PCNA, green) and the neuronal stem cell marker sox2 (red) on a single whole mount olfactory lamella. Two rows of dividing cells can be identified in the peripheral nonsensory OE, cells at the inner band (arrows) are double positive for sox2 and appear yellow, while dividing cells in the peripheral OE (arrowheads) are devoid of sox2 expression. Note that most of the double-positive dividing cells at the interlamellar curve (left end of the lamella) are not included due to the dissection of the lamella.

Because OSNs have a limited life span and little, if any, mitotic activity occurs in the sensory OE, cell division at the centre and the periphery of the OE must account for OSN neurogenesis. To investigate the potential contribution to the generation of neurons we examined tissue expression of the early neurogenesis markers deltaA (dla) and achaete-scute family bHLH transcription factor 1a (ascl1a), and the late OSN differentiation markers neuronal differentiation 4 (neurod4) and growth-associated protein 43 (gap43) by *in situ*-hybridization on OE cross sections (Fig. 4). *Mash1* (the mammalian orthologue of *ascl1a*) is the first proneural gene expressed by the globose basal progenitor population in the mouse OE^17^. *Ascl1a*-positive cells were sparse and formed small clusters exclusively at the ILC and the SNS, while other regions of the OE, including the most peripheral proliferation zone, were devoid of *ascl1* expression. Thus, *ascl1* expression colocalises with the ILC and SNS, suggesting that they have neurogenic potential. Of the average 30.3 ± 3.5 (mean ± SEM, 8 sections from 2 fish) cells per section, 31.3 ± 3.3% (mean ± SEM) were located at the ILC while twice as many, 67.0 ± 2.6%, were found at the SNS.

The balance between maintenance of multipotent precursor pools and the generation of committed neuronal lineages is regulated by notch/delta signalling with delta-positive cells typically developing into differentiated neurons^23^. *Dla*-expressing cells were equally sparse and exclusively found at the ILC and SNS. No additional *dla*-positive cells could be detected in other regions of the OE, including the peripheral nonsensory OE. On average 28.2 ± 1.9 (9 sections from 3 fish) *dla*-positive cells could be detected per section, 28.4 ± 3.5% and 69.2 ± 3.4% of which resided at the ILC and SNS, respectively. Thus, similar to *ascl1a*, about two times more *dla*-positive cells are present at the SNS than the ILC.

Simultaneous labelling of *asl1a* or *dla* by *in situ*-hybridization and BrdU staining revealed close positional overlap, including the presence of double-positive cells (Fig. 4B). Given the relatively low abundance of *ascl1a*- and *dla*-expressing cells this implies that they are rapidly cycling neuronal precursors, which actively contribute to OSN neurogenesis. The narrow expression patterns of *ascl1a* and *dla* suggests that only mitotic activity at the ILC and SNS is neurogenic, while the *ascl1a*-/*dla*-negative proliferation zone in the peripheral OE does not generate OSNs. The functional difference is further substantiated by whole mount immunohistochemistry for the mitotic marker proliferating cell nuclear antigen (PCNA) and the general neuronal stem cell marker sox2^24^. Only PCNA-positive cells at the ILC and SNS but not in the peripheral OE are positive for sox2 (Fig. 4D).

To investigate the distribution of later immediate neuronal precursors, the tissue expression of *neurod4* was analysed (Fig. 4). Contrary to early neuronal markers, *neurod4* had a higher propensity to be expressed at the ILC (55.2 ± 6.7%) than the SNS (40.6 ± 6.1%), while no additional *neurod4*-positive cells were found outside these regions. This may imply that either more neurons are produced at the ILC despite the higher abundance of *dla*- and *ascl1a* at the SNS, or that additional, yet unidentified, neuronal differentiation factors, such as neurod1 (S. Capar and S.H. Fuss, unpublished) assume a complementary role at the SNS, eventually to specify distinct neuronal lineages.

Growth-associated protein 43 (gap43) is a marker of immature OSNs^25^ and is required for axon outgrowth during OSN development. Neurogenesis in the rodent OE is followed by vertical migration of differentiating OSNs and gap43-positive cells are located at intermediate positions between basal progenitors and mature OSNs in the apical OE^17, 25^. In line with the radial displacement of cells in the zebrafish OE, *gap43*-positive cells were located predominantly at the ILC and sparsely at the SNS, yet, gap43 expression was more diffuse and extended further towards the inner sensory OE than *ascl1a* or *dla* (Fig. 4).

### Functional equivalence of ILC and SNS neurogenesis

A milestone in OSN development is the expression of specific chemosensory receptor genes, which initiates functional maturity. The single zebrafish OE contains morphologically distinct cell types equivalent to different chemosensory subsystems, such as the main olfactory and vomeronasal system. Ciliated OSNs express classical OR genes and the cellular marker *olfactory marker protein b* (*ompb*)^26, 27^ while microvillous cells express vomeronasal-like receptors and stain positive for the marker *transient receptor potential channel C2* (*trpc2*)^27^. The uneven distribution of neuronal differentiation markers raises the question as to whether the two neurogenic sites at the ILC and SNS are functionally equivalent or if they generate different OSN subtypes.

**Figure 5:**
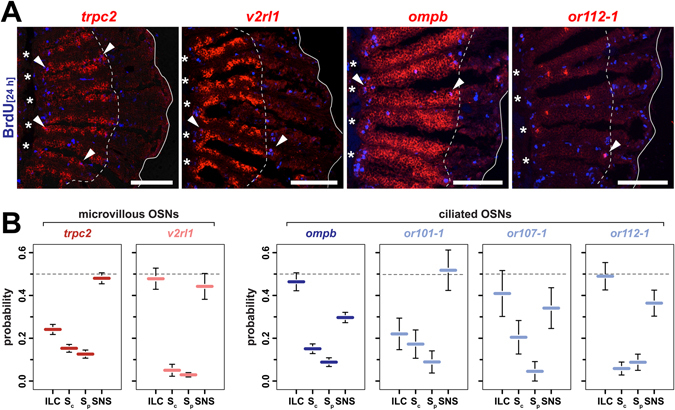
Functional equivalence and bias in OSN neurogenesis at the ILC and SNS. (**A**) *In situ*-hybridization (red) for markers of distinct OSN subtypes and immunohistochemistry against BrdU (blue) at 8d (*trpc2*, *ompb*) and 4d (*v2rl1*, *or112*-*1*) following 24 h BrdU incubation. The *trpc2* gene is expressed by all microvillous OSNs, a larger subset of which also expresses the vomeronasal receptor *v2rl1*. Ciliated OSNs label positive for *ompb* and express specific olfactory receptor genes, such as *or112*-*1*. For each gene, double-positive cells (arrowheads) can be found at the interlamellar curves (asterisks) and the sensory/nonsensory border (dotted line). The solid line demarcates the outline of the section of which only one half is shown. Scale bars: 100 µm. (**B**) Quantification of OSN subtype neurogenesis at different proliferation sites. The sensory OE was subdivided into four equal segments (ILC: interlamellar curves, Sc: sensory central, Sp: sensory peripheral, SNS: sensory/nonsensory border) and the occurrence of marker/BrdU double-positive OSNs was scored for each segment for the general microvillous marker *trpc2* (red), the vomeronasal-like receptor *v2rl1* (light red), the ciliated OSN marker *ompb* (blue) and the three classical OR genes *or101*-*1*, *or107*-*1*, and *or112*-*1* (light blue) expressed by ciliated OSNs. A subtype-specific bias exists for neurogenesis at the ILC and SNS. Data represent the mean ± SEM; *trpc2*: 607 cells from 5 OE; *v2rl1*: 203 cells from 4 OE; *ompb*: 563 cells from 2 OE; *or101*-*1*: 35 cells from 6 OE; *or107*: 25 cells from 7 OE; and *or112*-*1*: 76 cells from 8 OE.

To understand whether progenitor cells at the ILC and/or SNS can give rise to ciliated OSNs, zebrafish were incubated with BrdU for 24 h and analysed 7d later by *in situ*-hybridization against *ompb* and BrdU immunohistochemistry (Fig. 5). *Ompb*-expressing cells are uniformly distributed across the sensory OE without any apparent radial preference and occupy intermediate apico-basal layers. As expected from the positional shift of BrdU-positive cells over the 8d period, spatial overlap between BrdU-positive and *ompb*-expressing cells was observed at the edge of the sensory OE. Importantly, *ompb*/BrdU double-positive cells were found at both sites, suggesting that ciliated OSNs are generated at the ILC and the SNS.

To quantify the contribution of each proliferation zone to ciliated OSN neurogenesis, the radial positions of *ompb*/BrdU double-positive cells were categorized by dividing the sensory OE into four segments of equal length (ILC, sensory central, sensory peripheral, SNS). In total, 46.4 ± 4.2% (mean ± SEM, 10 sections from 2 OEs, 563 cells) of BrdU/*ompb* double-positive cells were located in the ILC segment, whereas only 29.7 ± 2.4% of cells were found close to the SNS. A smaller fraction of 15.1 ± 2.3% and 8.8 ± 2.0% *ompb*/BrdU double-positive cells were found in the central and peripheral sensory OE, respectively (Fig. 5B), which may have originated from the ILC and SNS but invaded the OE during the 8d period. Therefore, a total of 61% of ciliated OSNs may be ILC-derived and 39% may have been generated at the SNS. Next, we performed a similar analysis for *trpc2*-positive microvillous OSNs, of which 39% were generated at the ILC and 61% derived from the SNS (25 sections from 5 fish, 607 cells). Thus, no fundamental difference exists between the ILC and the SNS with respect to the generation of the major morphological OSN subtypes, yet, a clear bias for the generation of ciliated OSNs at the ILC and for the generation of microvillous cells at the SNS was observed. Curiously, the observation that most *ompb*/BrdU and *trpc2*/BrdU double-positive OSNs were found close to the proliferation zones suggests that functional maturation of OSNs precedes completion of positional shifts across the OE.

In total there are 176 ORs and 58 vomeronasal receptors a developing zebrafish OSN could choose to express^28–30^. To understand whether systematic differences occur for the generation of specific OSNs subsets, we extended the BrdU-birthdating analysis to individual chemoreceptor genes (Fig. 5). OSNs expressing either one of the highly expressed vomeronasal receptor *v2rl1* or the less abundantly expressed classical OR genes *or101*-*1*, *or107*-*1*, and *or112*-*1* originated from both proliferation zones but again with receptor-specific spatial bias.

V*2rl1*/BrdU-expressing cells were evenly generated from the ILC and SNS with 53.2% and 46.8%, respectively (4 complete OEs, 203 cells, Fig. 5B). Interestingly, the 1.1 ratio of cells generated at the ILC relative to the SNS was different from the ratio of 0.6 for *trpc2*-positive microvillous cells of which *v2rl1*-expressing cells are a subset. For the classical OR genes 1.2-fold more *or112*-*1*-expressing cells (ILC: 54.8%, SNS45.2%; 8 OEs, 76 cells) and 1.6-times more *or107*-*1*-positive cells (ILC: 61.4%, SNS: 38.6%; 7 OEs, 25 cells) were generated at the ILC, whereas only a ILC to SNS ratio of 0.6 was found for the generation of *or101*-*1*-expressing cells (ILC: 39.3%, SNS: 60.7%; 6 OEs, 35 cells). Thus, all OSN subpopulations analysed here originated from both neurogenic niches at the ILC and SNS. However, the observed bias suggests an unequal distribution of lineage-restricted stem/progenitor populations or of factors that affect the niches post-mitotically to induce distinct developmental fates.

### OR expression patterns are established over time

The pattern of OR gene expression in zebrafish has been described as non-random^11^ in the way that OSNs expressing different OR genes occupy preferred concentric domains that are reminiscent of zones. OR-specific expression domains could arise from at least three different mechanisms. OSNs generated at distant neurogenic sites may migrate radially along the OE until they encounter positional signals that promote expression of defined OR subsets. However, this is unlikely because OSNs turn on OR expression while they are still close to the edges of the sensory OE (Fig. 5), thus, before or while they are undergoing positional shifts. Alternatively, OSNs may stop their movement at OE positions defined by specific molecular signals, or they may undergo (OR-specific) cell death while invading the OE, thereby generating the impression of defined ring-like expression domains.

**Figure 6:**
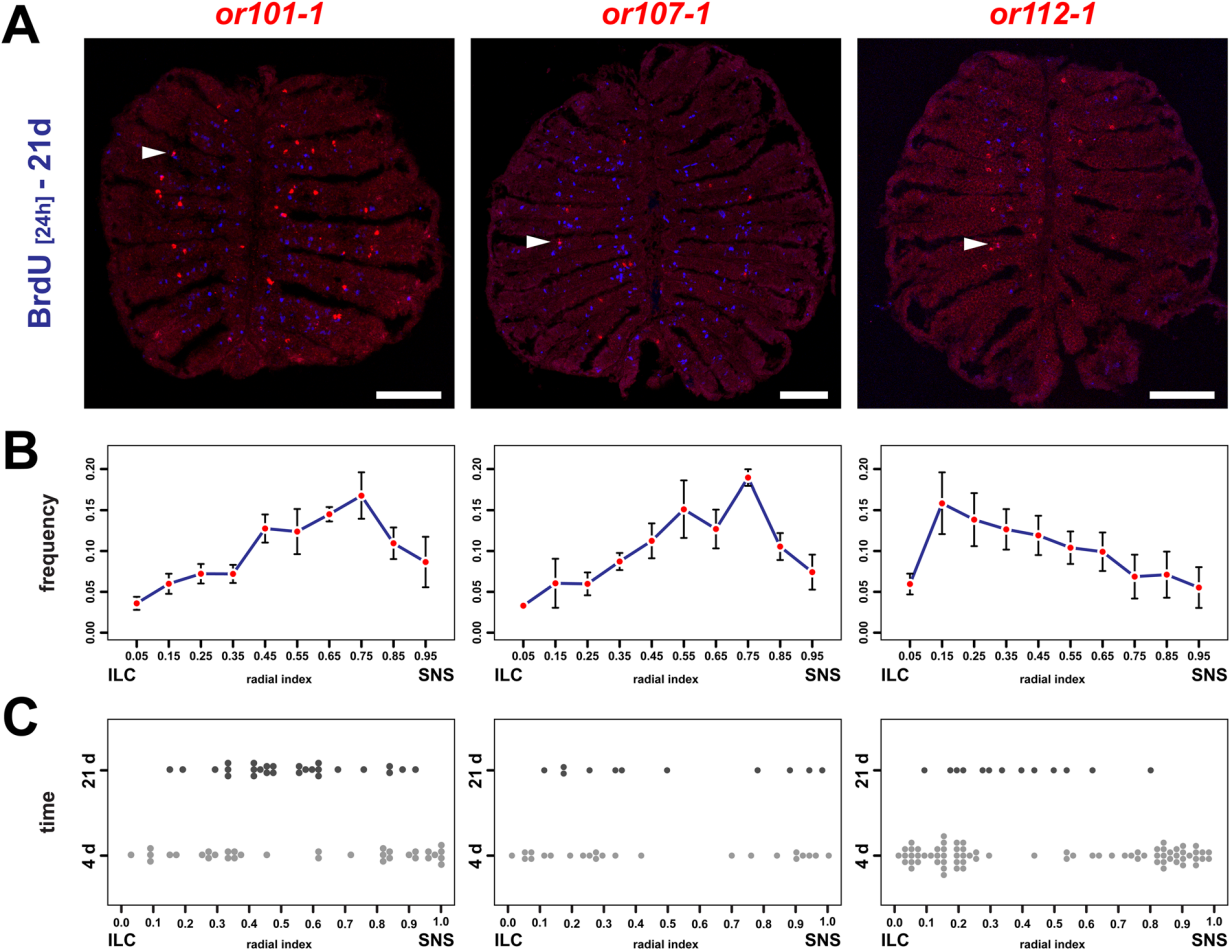
OSNs continue to undergo positional shifts after onset of OR expression. (**A**) Immunohistochemistry against BrdU (blue) on cross sections through the OE of adult zebrafish that were incubated for 24 h in BrdU 21d prior to analysis and simultaneous *in situ*-hybridization (red) for the three olfactory receptors *or101*-*1* (left), *or107*-*1* (centre), and *or112*-*1* (right). For each receptor BrdU double-positive cells (arrowheads) can be identified. Scale bar: 100 µm. (**B**) OR frequency distribution profiles across the radial dimension of the OE for the receptors shown in A (left: *or101*-*1*, centre: *or107*-*1*, right: *or112*-*1*). The position of each cell was normalized to the distance of the sensory/nonsensory border (SNS) from the tips of the interlamellar curves (ILCs). The data represent the positions (mean ± SEM) of: *or101*-*1*: 755 cells from 4 OEs of 4 fish, *or107*-*1*: 332 cells from 4 OEs of 4 fish, and *or112*-*1*: 464 cells from 6 OEs of 3 fish. (**C**) Spatial distribution of young (4d, light grey) and old (21d, dark grey) OSNs expressing *or101*-*1* (left), *or 107*-*1* (centre), and *or112*-*1* (right) normalized to the length of the sensory OE between the tips of the interlamellar curves (ILCs) and the sensory/nonsensory border (SNS). OSNs expressing specific OR genes continue to undergo positional shifts over time.

To discriminate between the latter possibilities, the positions of “young” and “old” OR-specific OSN subpopulations were analysed 4d and 21d after BrdU incubation, with respect to the overall expression profile of the OR gene (Fig. 6). The *or101*-*1*, *or107*-*1*, and *or112*-*1* genes were previously shown to occupy peripheral, intermediate, and central expression domains, respectively^11^. Plotting their frequency distribution over radial positions (Fig. 6B), in principle, confirmed this observation and a specific peak distribution could be observed for each OR. Importantly, and in line with our observation of radial progression of OSNs, cells expressing either one of the three ORs were also present all along the radial extent of the sensory OE. Thus, OR expression patterns in zebrafish are fundamentally different from the sharply delineated zones in the rodent OE^12^. Interestingly, all three OR profiles were bimodal and consisted of a major peak at one end of the OE and a shoulder distribution at the opposite end of the OE.

When the radial positions of OSNs expressing *or101*-*1*, *or107*-*1*, and *or112*-*1* that were generated 4d or 21d prior to analysis were plotted together (Fig. 6C), the younger populations had bimodal distributions with cells clustered at both ends of the OE while the older populations occupied more central positions that correlated loosely, but not perfectly, with local maxima of the OR expression patterns. Thus, OR-expressing OSNs continue to undergo positional shifts as they age. This notwithstanding, even the older OSN populations were rather broadly distributed, arguing against the presence of positional molecular markers in the tissue to direct active OSN migration to specific positions. Interestingly, we noticed that the overall likelihood of detecting *or*/BrdU double-positive OSNs decreased by 34% to 50% for the older population suggesting that zebrafish OSN may have a relatively short half life of about 17d to 25d.

#### Zebrafish OSNs have a limited life span

**Figure 7:**
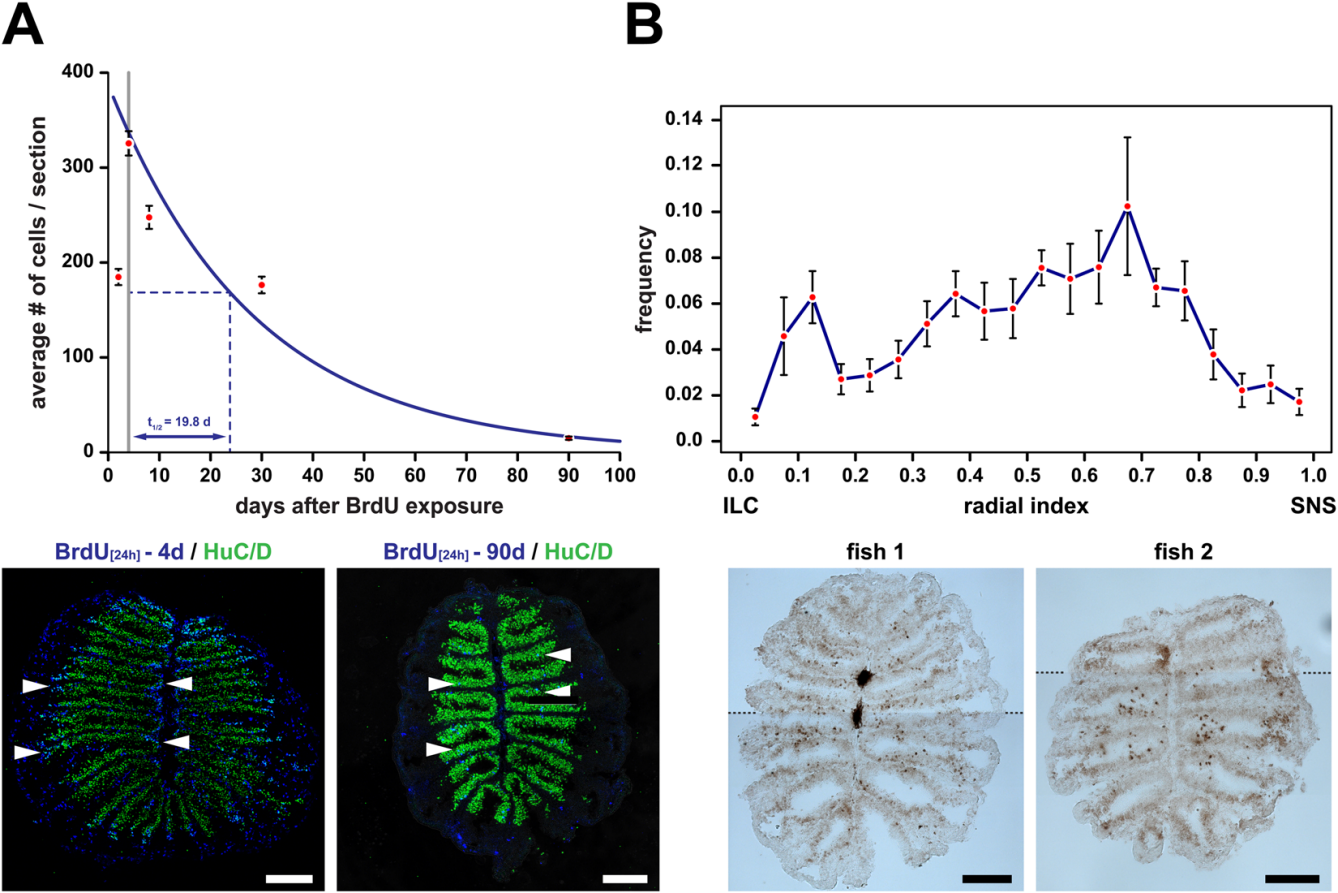
Cell death in the zebrafish OE. (**A**) Immunohistochemistry against BrdU and HuC/D and survival curve of BrdU/HuC double-positive OSNs (top; mean ± SEM; top) following 24 h incubation with BrdU. The data points after the initial increase of double positive cells between 2d and 4d can be fitted with the exponential function #cells = 387.5e^−0.035 X^, which corresponds to an OSN half life of t_1/2_ = 19.8 d. (n = 2d: 17, 4d: 26, 8d: 18, 30d: 51, and 90d: 60 sections). Representative cross sections for the 4d (left) and 90d (right) time point are shown (bottom). BrdU/HuC double-positive cells (turquoise, arrowheads) are more numerous at earlier time points and undergo positional shifts towards the centre of the sensory OE. (**B**) Detection of cell death in the adult zebrafish OE by terminal deoxynucleotidyl transferase dUTP nick end labeling (TUNEL) of fragmented DNA on cross sections though the OEs of two different zebrafish (bottom). TUNEL-positive cells appear dark brown by chromogenic detection with 3,3′-diaminobenzidine. The images represent composites of two high-resolution pictures joined together at indicated positions (dotted lines) to cover the full sections. The frequency distribution of TUNEL-positive cells along the radial dimension of the sensory region of the OE is shown in the top graph. The position of cells has been normalized to the distance of the sensory/nonsensory border (SNS) from the tip of the interlamellar curve (ILC). TUNEL-positive cells can be found throughout the entire sensory OE but are unevenly distributed and more abundant in the vicinity of the ILC and in the centre of the sensory OE. The data points represent the means ± SEM of the distribution of 689 cells from 9 sections of 3 fish. Scale bars: 100 µm.

To more accurately determine the average life span of zebrafish OSNs, we incubated adult fish in BrdU for 24 h and analysed the OE for BrdU and HuC/D immunoreactivity 2d, 4d, 8d, 30d, and 90d following treatment (Fig. 7A). Sections were scored for the number of BrdU/HuC double-positive cells and plotted over time. Interestingly, the highest number of double-positive cells was found 4d after BrdU incubation, suggesting that full OSN maturation may take between 3d to 5d. The data can be fitted by the exponential equation #cells = 387.5e^−0.035 x^ (R^2^: 0.98), which is equivalent to an OSN half life of 19.8d days or a mean OSN life span of 28.6d. Thus, zebrafish OSNs have a comparably short life span, which is about 4-fold shorter than the lifetime of mouse OSNs^16^. Curiously, the mean lifetime of 29d would allow an OSN to traverse exactly half the OE at the determined average speed of 0.017 radial indices/d (Supplementary Fig. 3).

To investigate the elimination of OSNs from the OE more directly we stained OE cross sections by immunohistochemistry against the apoptotic marker cleaved caspase 3 but could detect only a very low number of apoptotic cells (Supplementary Fig. 4). To control for the reactivity of the antiserum we chemically induced degeneration of the OE^21^, which resulted in a concomitant induction of caspase-positive cells around lesioned sites, suggesting that apoptotic OSNs in the intact OE are removed very rapidly from the OE or that OSNs are eliminated by a caspase-independent mechanism. Examination of cell death by terminal deoxynucleotidyl transferase dUTP nick end labelling (TUNEL), which may detect alternative forms of cell death, such as necrosis or autolytic cell death^31^, revealed a much larger number of dying cells (Fig. 7B). When we plotted the distribution of TUNEL-positive cells across the sensory OE we observed an accumulation of cells in the centre of the OE consistent with the observation that OSNs undergo positional shifts and that older cells progressively occupy positions in the mid OE. Interestingly, a second peak was observed close to the ILC, suggesting that a substantial number of adult-born cells may fail to develop and is eliminated shortly after generation.

#### A computational model for steady-state OR expression profiles

How can continuously moving OSNs generate a seemingly specific and positionally stable OR expression pattern? Our study provided key descriptors of OSN neurogenesis, such as positional bias of OSN generation at the ILC and SNS, the average life span of OSNs, and a rough estimate of the OSN maturation time of about 4d. We estimated additional parameters from the linear mixture models (Supplementary Fig. 3) to understand if the combination of these simple properties of OSN neurogenesis and cell migration can accurately describe OR expression patterns. To simplify parameter approximation a Gaussian normal (in addition to the lognormal) model was generated. The positional changes of the moving peaks can be described by a linear regression line with parameters: ILC → SNS: y = 0.0101x + 0.0822 (R^2^: 0.99) and SNS → ILC: y = 0.0169x + 1.169 (R^2^: 1.0). In other words, OSNs shift centrifugally from the ILC with an average speed of 0.01 radial indices/d, while OSNs from the SNS migrates centripetally and with a speed of 0.017 radial indices/d (Supplementary Fig. 3B,C). A similar estimation was performed for the changing dispersions of OSN distributions (σ), which become broader as OSNs invade the sensory OE (Supplementary Fig. 3D). The best fit was obtained with ∆σ_ILC_ = 0.0024x + 0.0701 (R^2^: 0.99), which indicates that the standard deviation of the approximated normal distribution grows by 0.0024 σ/d.

**Figure 8:**
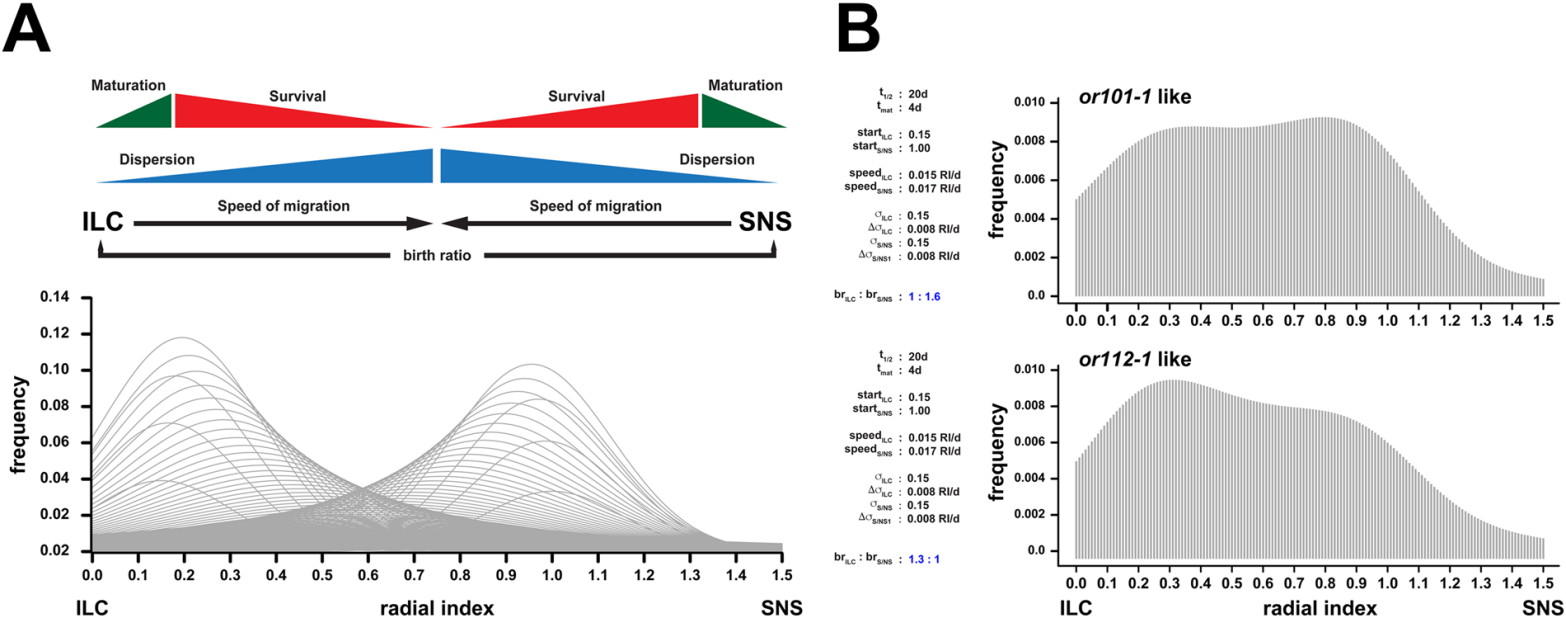
A mathematical model for OR expression profiles. (**A**) Architecture of the mathematical model developed to describe the generation of specific OR gene expression patterns in the zebrafish OE. The model function generates OSN distribution profiles for consecutive days based on their time to maturation, half life, speed of positional shifts across the OE, increase in dispersion (σ), and the bias in generation at the interlamellar curve (ILC) and sensory/nonsensory border (SNS). The resulting OR profile is equivalent to the positional sum of the timed distributions. The full R code of the model can be found in the Supplementary Material. (**B**) Model output for two different parameter sets to model *or101*-*1*- (top) *and or112*-*1*-like (bottom) distributions, which represent the two extremes analysed in this study. The *or101*-*1*-like profile can be changed into an *or112*-*1*-like distribution by adjusting the bias in OSN generation at the ILC and SNS (br_ILC_: br_SNS_: birth ratio ILC: SNS, blue). A more detailed description of the model output and its dependency on the various parameters is given in the Supplementary Material.

A mathematical model was then generated in R^32^ based on the quantitative parameters described above (Fig. 8A, see Supplementary Material for the full R code of the model). At its core, the model generates the positional sums of the spatial distribution profiles of differently aged cell populations, taking into account the shift in position of the peak for each day, the increase in dispersion over time, and the reduction in cell number according to OSN half life.

The model generated spatial profiles that closely resemble the observed expression profiles of the different OR-expressing OSN populations. For instance, when parameters derived from *or112*-*1* were fed into the model a profile similar to the distribution of *or112*-*1* was obtained (Fig. 8B, Supplementary Fig. 5), whereas if specific *or101*-*1* parameters were used, a more *or101*-*1*-like profile was generated. The model is most sensitive to changes in lifetime of OSN populations and positional bias in neurogenesis, whereas changes in σ and modest changes in velocity affected the outcome less severely (Supplementary Fig. 5). Most importantly, however, the model generates OR-like distribution patterns without the need to invoke additional parameters, including molecular cues to set up defined molecular domains within the zebrafish OE.

## Discussion

Structured OR expression patterns are common across species but may differ widely in their spatial definition, function, and developmental origin. Because OR expression is inherently linked to ontogenetic OSN development we studied adult neurogenesis to understand the generation of OR expression domains in the zebrafish OE. We find that in the adult OE new OSNs are generated from progenitor cells located exclusively at the ILC and SNS and that other regions of the OE, including the sensory OE, do not contribute to OSN neurogenesis. Interestingly, OSNs reach functional maturity rapidly and close to the site where they were generated but subsequently invade the sensory OE by continuous and life-long radial shifts in position, suggesting that the tissue does not provide molecular signals to specify distinct OR expression domains. In fact, the establishment of OR-specific expression patterns can be explained accurately by a limited set of non-molecular parameters, such as biased generation of OSN subpopulations at the ILC and SNS, OSN maturation time, the velocity of movement across the OE, and OSN lifetime.

In rodents, OSNs originate from globose basal cells, which form a heterogeneous cell population in the basal OE that includes multipotent stem/progenitor cells, transit amplifying cells, and immediate neuronal precursors^17^. The transition from progenitors cells to mature OSNs can be followed by successive expression of molecular markers concomitant with basal to apical movement^18^. We find that OSNs in zebrafish migrate radially, rather than vertically. Consistently, the globose basal cell markers *ascl1a*, *dla*, and *neurod1/4* are expressed at the ILC and SNS, while markers for immature (*gap43*) and mature OSNs (*ompb*, *trpc2*, *ORs*) are expressed at increasing distance. Thus, neurogenesis in the fish OE more closely resembles neurogenesis in the rodent vomeronasal organ in which neurons are generated at the marginal zones and invade the neuroepithelium over time^33, 34^.

The basal lamina in the centre of lamellae could be a suitable substrate for active OSN migration. Yet, even though BrdU-positive cells at the ILC and SNS had a tendency to be more basal, we could not observe this for moving cells, suggesting that other mechanisms generate positional shifts of ageing OSN populations. For instance, primordial germ cells migrate long distances in larval zebrafish using transient cell-cell interactions as scaffold for directional movement towards local guidance cues^35^. In either case, attractive guidance signals would need to be positioned in the mid OE or repulsive signals in the ILC and SNS. Yet, the positions of cells shifted only for about 5 μm, or half a cell diameter per day. Thus, a more parsimonious mechanism may be passive displacement by successively born OSNs. It is striking that the observed movement would allow an OSN to traverse exactly half of the sensory OE at the estimated average OSN life span of 29 d. We observed increased cell death in the centre of the sensory OE consistent with an accumulation of aged cells. It remains to be seen in as much elimination of aged OSNs in the centre of the OE may contribute to the dynamics of positional shifts while it is understandable that the process allows incoming newly generated cells to integrate into the tissue without substantial progressive growth of the OE.

According to our cell fate analysis, no fundamental functional difference exists between the ILC and SNS as microvillous and ciliated OSNs originated from both zones, so did OR-specific OSN subpopulations. Even though ILC and SNS are distant from each other in the dorsal OE they are continuous in the ventral epithelium and may have a common developmental origin. It appears that the SNS is more active overall as a higher number of BrdU- and pH3-positive cells were found there, however, it was impossible to deduce straight-forward rules as to which subpopulations are generated at which site. Almost twice as many ciliated OSNs were generated at the ILC, whereas an opposite bias existed for microvillous cells. It is curious, however, that cells expressing specific receptors did not follow the exact same bias as the class of OSN by which they are expressed. For instance, *v2rl1*-expressing microvillous OSNs were born at equal rates at the ILC and SNS contrary to the entire population of *trpc2*-positive microvillous cells. Similarly, *or112*-*1*- and *or107*-*1*-expressing OSNs were generated preferentially at the ILC, while more *or101*-*1* cells originated from the SNS. Our model of OSN dynamics shows that biased generation at the centre or periphery of the OE is a major determinant of the resulting OR distribution pattern. A likely explanation for the biased production of OSN subpopulations may be the uneven distribution of lineage-restricted progenitors, a phenomenon that is not well understood but immortalized OSN cell lines have been reported to generate restricted OR subrepertoires in a non-clonal fashion *in vitro* ^36, 37^. More recently, a mechanism for OR gene choice has been proposed based on epigenetic changes at OR gene promoters and trans-interaction of OR enhancer elements^3^. Yet, this mechanism has not yet been linked to OR repertoire restriction or zonal expression. Regardless of the exact molecular mechanism, the ILC and SNS are largely equivalent stem cell niches and do not represent fundamentally different progenitor pools.

Another critical parameter in our model is the short lifetime of zebrafish OSNs of 29 days. Longer and shorter half-lives severely distorted the pattern and rendered it less OR-like. Rodent OSNs have up to five-fold longer lifetimes^15, 16^, which is affected by odorant sensitivity^38, 39^. The relative expression frequencies of the three OR genes studied here (or112-1 < or107-1 < or101-1), however, do not correlate with the specific differences in the calculated life spans of OSNs expressing these ORs, suggesting that the relative frequencies are determined by a combination of generation rate and selective survival.

The existence of zonal OR expression patterns raises the question as to what their functional significance, if any, may be. It could serve a true role in odour detection or could be a less well understood aspect of the OR gene choice mechanism by which an OSN ensures that it expresses only one of the many different ORs. The multitude and diversity of OR genes, up to several thousands in some species^40^, has evolved from multiple gene duplication and diversification events. Thus, it is conceivable that ORs with similar coding sequence and, therefore, related response profiles are responsive to the same regulatory factors active in the same OE zone. Consequently, they would form glomeruli in neighbouring positions in the olfactory bulb and enable lateral processing of glomeruli receiving related sensory input to increase odour discrimination. The sorption hypothesis states that physicochemical features of chemicals and the aerodynamic properties of the nose affect the likelihood with which a chemical ligand can interact effectively with a receptor^41^, a hypothesis that is plausible when odorants have to transition from the airphase into the aqueous phase before interacting with the receptor. This, however, does not occur in aquatic species, such as zebrafish, where the odorants are already dissolved in water.

Even though, we provide some evidence that the apparent organized OR expression pattern can be generated without the need to pattern the tissue with morphogenes, we cannot entirely exclude molecular influences. For instance the biased generation of OSNs at the ILC and SNS certainly has molecular underpinnings, so does the observation that OSNs derived from the SNS never shift towards the peripheral end of the OE. However, the continuous movement of OSNs until they are eliminated from the OE strongly argues that positional cues do not exist. If anything, the rather even distribution OSNs expressing specific ORs would make the surface of the OE equally sensitive to odorants. For the same reason, the hypotheses that the precise position of OSNs in the zebrafish OE is related to OR gene choice or would affect glomerular positions in the olfactory bulb can be excluded.

A growing body of data shows that even complex, highly specific, and stereotyped developmental patterns can be, at least in part, generated by stochastic and cell-autonomous behaviours that depend on the mutual influences of neighbouring cells and tissue dynamics^42^. Examples include, but are not limited to, retinal development^43^, sensory projections to the brain^44^, synaptic connections and neuronal patterning^45^. The combination of dynamic, largely stochastic and cell-autonomous principles that patterns the zebrafish OE represents another example of how stereotyped outcomes can be generated without the need of precisely specifying each single developmental step.

## Methods

### Animals

Adult (>6 months of age) zebrafish (*Danio rerio*) of the AB/AB laboratory strain were maintained at the research animal facility of Boğaziçi University Center for Life Sciences and Technologies. Animals were kept at a density of 5 fish/l in 10-liter tanks at 28 ± 2 °C temperature, 14 h/10 h light dark cycles, with five-stage filtration, aeration, and UV sterilization (Aquatic Habitats, FL, USA). Artificial fresh water was prepared by dissolving 2.0 g sea salt, 7.5 g sodium bicarbonate, and 0.84 g calcium sulphate in 100 l of reverse osmosis water. Fish were fed twice a day with flake food supplemented once with live brine shrimp larvae (*Artemia sp*.). The use of experimental zebrafish for this study (Olfactory neurogenesis during tissue maintenance and repair) has been approved by the Institutional Ethics Board for Animal Experiments at Bogazici University (BÜHADYEK) under title 2012-11-28 and all experiments and methods were performed in accordance with relevant guidelines and regulations including the National Animal Protection Act (law number 5199, published on 24.06.2004) and the directive 2010/63/EU of the European Parliament and of the Council of 22. September 2010 on the protection of animals used for scientific purposes.

### Cell proliferation assays

In order to detect proliferating cells, zebrafish (>6 months of age) were incubated for 24 hours in tank water containing 30 mg/l of the halogenated thymidine analog 5-Bromo-2′-deoxyuridine (BrdU) in the dark at 28 °C. Olfactory epithelia were dissected out, cryosectioned at 12 µm thickness and fixed with a 3:7 mixture (vol/vol) glycine-HCl: absolute EtOH for 45 min at room temperature. Slides were washed 3 times with 1x PBS (137 mM NaCl, 2.7 mM KCl, 10 mM Na_2_HPO_4_, pH: 7.4) and incubated with 4 M HCl for 15 min to permeabilize nuclei and denature DNA. Following 3 washes in 1x PBS, slides were incubated for 1 h in blocking solution (0.8% BSA, 10% goat or donkey serum in 1X PBT) for one hour followed by overnight treatment with mouse anti-BrdU (Becton Dickinson, USA; clone B44) or rat anti-BrdU (Abcam, UK; clone BU1/75) primary antibodies (1:250 in blocking solution) at 4 °C. Cells were visualized by subsequent detection with either anti-mouse Alexa-555 or anti-rat Alexa-637 (Thermo Fisher Scientific, USA) secondary antibodies (1:800 in blocking solution) for 2 h at room temperature.

For labelling of differently aged cell populations, fish were first incubated in 30 mg/l 5-Iodo-2′-deoxyuridine (IdU) for 24 h, transferred to fresh tank water at least 3 times to remove any residual IdU and kept in clean water for 2d or 6d until a second 24 h incubation in 30 mg/l 5-Chloro-2′-deoxyuridine. Sections were first incubated with mouse anti-BrdU (Becton Dickinson, USA; clone B44) primary antibody overnight at 4 °C to detect cells that incorporated IdU, rinsed with 1x PBS, followed by high stringency washes in TBST buffer at 37 °C while shaking at 220 rpm for 1 h to remove any residual antibody^46^. Samples were then processed with rat anti-BrdU antibody (Abcam, UK; clone BU1/75) overnight at 4 °C to detect incorporated CldU and visualized with anti mouse Alexa-555 an anti-rat Alexa 637 secondary antibodies for 2 hours.

### Gene expression analysis by *in situ*-hybridization

*In situ* hybridization using appropriate antisense riboprobes against target genes was performed as described elsewhere^47^. Briefly, OE tissue sections were fixed in 4% paraformaldehyde (pH: 7.4) for 10 min at room temperature, washed with 1x PBS for 5 min, incubated in 0.2 M HCl for 10 min and washed in 1x PBS again. The tissue was treated with proteinase K (2 μl in 40 ml 0.1 M Tris-HCl, pH: 8.0) for 7.5 min at 37 °C, washed in 1x PBS, and incubated in 1% tri-ethylamine for 10 min and washed again in 1x PBS. Slides were hybridized over night at 66–70 °C with 3 µg/ml DIG-labeled antisense riboprobe in hybridization buffer (50% deionized formamide, 5x SSC, 0.1% Tween 20, 50 mg/ml heparin, 500 mg/ml RNase-free tRNA, pH 6.0). Post-hybridization washes were performed in a decreasing series of sodium-sodium citrate concentrations (75%, 50%, 25% 0.2X SSC in 1x PBS) at the hybridization temperature. Slides were blocked in 0.5% blocking solution (Roche) and incubated with anti-DIG antibody (Roche; 1:750 in 0.5% blocking solution). For detection, slides were washed in 1x PBS for 15 min each and pre-incubated in detection buffer before HNPP detection (Roche) for 30 min to 2 h depending on the strength of the signal.

Templates used to generate antisense riboprobes correspond to exonic sequences of the following genes that were amplified from zebrafish OE cDNA using the listed oligonucleotide primers: *ascl1a*: 589 bp fragment of ENSDARG00000038386 between ascl1a-F: 5′-GACATCACCGCCAAGATGGAAA-3′ and ascl1a-R: 5′-TCAAAACCAGTTGGTGAAGTCCAG-3′, dla: 559 bp fragment of ENSDARG00000010791 between dla-F: 5′-TCGTTCAAAGGTTCAGCAGCGAC-3′ and dla-R: 5′-CGTGGATGCCGATGCACATGCCGAC-3′; neurod4: 533 bp fragment of ENSDARG00000003469 between neurod4-F: 5-GCAAGACAAGAACGTTTCCGTGCC-3′ and neurod4-R: 5′-ATGCTGAGTGGTGGAGTTAGAGG-3′; trpc2b: 608 bp fragment of ENSDARG00000003344, between trpc2b-F: 5′-CACTCGATTGGCATACATCCTTCCA-3′ and trpc2b-R: 5′-TGCGGTTTTCTCCTGAGCCTCCTT-3′; v2rl1: 634 bp fragment of ENSDARG00000005942 between v2rl1-F: 5′-CGAGGATGACTATGGGAAATACGGC-3′ and v2rl1-R: 5′-AGCACATGTGTCGTTCTCAAAAGGCC-3′; or112-1: 750 bp fragment of ENSDARG00000077211 between or112-1-F: 5′-ATGACTGAGGAACTCCAAGGAGCA-3′ and or112-1-R: 5′-GACCATTAAGTGTGTACCACAGGTATGAAA-3; or107-1: 716 bp fragment of ENSDARG00000041032 between or107-1-F: 5′-CAGTCACATTCAGCAATGGAACGG-3′ and or107-1-R: 5′-GGCCTTCAGCGCTGGGTTACAG-3′; or101-1: 678 bp fragment of ENSDARG00000013014 between or101-1-F: 5′-ATGAACACCAGCGGCTCGGT-3′ and or101-1-R: 5′-GTGCCATGCTCATCCTTCTCA-3′; gap43: fragment of ENSDARG00000099744 between gap43-F: 5′-AAACCGGAGGAAAACGCTCA-3′ and 5′-TTAAACACTCTCCTCTGTGCCGG-3′; ompb: 1.148 bp full length cDNA clone^26^ of ENSDARG00000032380.

### Immunohistochemistry

For whole-mount immunohistochemistry zebrafish OE were dissected out in ice cold 1x PBS, fixed in 4% PFA for 3 hours at room temperature, rinsed 2x and washed 3x for 10 mins in PBST (1% v/v Triton X-100 in 1x PBS). To aid permeabilisation, OEs were transferred gradually to methanol by 1 × 5 min 50% MeOH/50% PBST and 3 × 5 min 100% MeOH washes and stored overnight at −20 °C. OE were then rehydrated by 1 × 5 min 50% MeOH/50% PBST and 3 × 5 min PBST washes. The OE were further permeabilised by treatment with Proteinase K (20 mg/ml, 30 U/mg Proteinase K diluted to 1:1000 in PBST) for 1 min at room temperature followed by 3x washes in PBST, before being transferred to 4% PFA for 20 min at room temperature for post fixation. Post fixed OEs were washed 3x in PBST, blocked with 3% BSA in 1x PBS for 1 h at room temperature, and incubated with primary rabbit anti-Sox2 (1:500, Genetex) and mouse anti-PCNA (1:500, Sigma) antibodies in blocking solution overnight at 4 °C. The OEs were washed rigorously in 1x PBST and incubated with secondary antibodies (1:200, anti-mouse Alexa-488; 1:200, anti-rabbit Alexa-647) over night at 4 °C. Immunostained OEs were mounted in 1% low-melting agarose for imaging purposes.

Frozen OE sections are fixed in 4% PFA for 10 minutes at room temperature, washed 3 times with 1x PBST, blcked in 3% BSA in 1x PBST for 1 h before incubation with mouse anti-HuC/D (1:500, Invitrogen), rabbit anti-phospho-histone H3 (Ser10; 1:250, Millipore), rabbit anti activated caspase-3 (1:100; BD Pharmingen) primary antibody at 4 °C over night. Samples were washed 3 times with PBS, followed by secondary antibody incubation (Alexa 488, 555, 647 anti-mouse/rabbit) for 1,5-2 hours at room temperature and sored in PBS.

### TUNEL assay

For detection of cell death, terminal deoxynucleotidyl transferase dUTP nick end labeling was performed using the ApopTag Plus Peroxidase *in situ* apoptosis kit (Millipore). Briefly, freshly dissected OE tissue was cryosectioned, fixed in 1% PFA/1x PBS for 10 min, followed by blocking of endogenous peroxidase activity in 3% H_2_O_2_/1x PBS for 30 min. Samples were subjected to the TdT reaction for 1 h at 37 °C, followed by immunohistochemistry against digoxigenin-labeled DNA ends, and cells were visualized by chromogenic conversion of 3,3′-diaminobenzidine.

### Image acquisition and processing

Tissue samples stained by immunohistochemistry and *in situ*-hybridization were visualized on a Leica SP5-AOBS confocal microscope using 20x or 40x lens magnification at 1.024 × 1.024 or 2.048 × 2.048 pixel resolution. Images were zoomed to fit entire OE cross-sections onto the image frame. Images were further processed in Leica LAS-AF (Leica, Germany) or FIJI^48^ image processing software by adjusting brightness and contrast, or cropping image frames.

### Morphometrical measurements

To enable quantitative descriptions of cell positions within the OE a spatial reference system based on the length and geometry of individual lamellae was developed (Fig. 1A). The radial position of each cell was determined as orthogonal projection of the cell’s position onto a straight line connecting the base of the interlamellar curve to the peripheral end of the OE along the lamella on which the cell was detected using LEICA-LAS-AF (Leica, Germany) or FIJI^48^ image processing software. Cellular positions were normalized to the entire length of the lamella or the distance of the SNS from the base of the ILC. Linear unmixing of compound distributions were performed using the mixdist^49^ package in R^32^.

### Generation of heatmaps/standard lamellae

To visualize the pattern of proliferating cells onto reconstructed side views of olfactory lamellae, the radial distribution profiles of BrdU-positive cells for 25 to 26 consecutive cross sections through the same olfactory epithelium were determined, normalized to the maximum number of cells per bin multiplied by 255 and visualized as text image in FIJI using the physics look up table. To smoothen the resulting image for visualization purposes the images were scaled 40-fold by a bicubic function in FIJI.

## Electronic supplementary material

Supplementary Material
