# Supplementary material for: Patterned Arrangements of Olfactory Receptor Gene Expression in Zebrafish are Established by Radial Movement of Specified Olfactory Sensory Neurons
Source: Sci Rep. 2017 Jul 17;7:5572. doi: 10.1038/s41598-017-06041-1 (PMC5514040; doi:10.1038/s41598-017-06041-1)
Supplement: Supplementary file 1 — Supplementary Material [file 41598_2017_6041_MOESM1_ESM.pdf]

Supplementary Material to

Patterned Arrangements of Olfactory Receptor Gene Expression in Zebrafish are Established by Radial Movement of Specified Olfactory Sensory Neurons

Xalid Bayramli[1], Yigit Kocagöz[1], Ugurcan Sakizli[1], and Stefan H. Fuss[1,2]

Author Affiliations:

[1] Bogaziçi University
Department of Molecular Biology and Genetics
34342 Bebek – Istanbul, Turkey

[2] Center for Life Sciences and Technologies
34342 Bebek – Istanbul, Turkey

Corresponding Author:
Stefan H. Fuss
Bogaziçi University
Department of Molecular Biology and Genetics
Kuzey Park 315
34342 Bebek – Istanbul, Turkey
phone: +90 212 359 7229
fax: +90 212 287 2468
email: stefan.fuss@boun.edu.tr

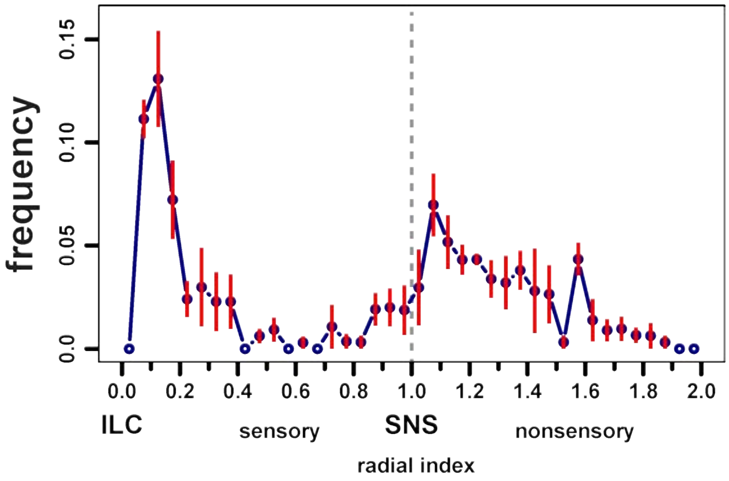

Supplementary Figure 1: Radial distribution of phospho-histone H3-positive cells.

Frequency distribution (mean ± SEM) of 266 phospho-histone H3-positive cells from 4 adult olfactory epithelia of 3 individual zebrafish plotted as function of their radial position along olfactory lamellae. The position of each cells was normalized to the distance of the sensory/nonsensory border (SNS, dotted grey line) from the tip of the interlamellar curve (ILC) for the lamella on which the cell was found. Mitotic cells show a high density at the ILC and just peripheral to the SNS, while the center of the sensory OE is largely devoid of acutely dividing cells.

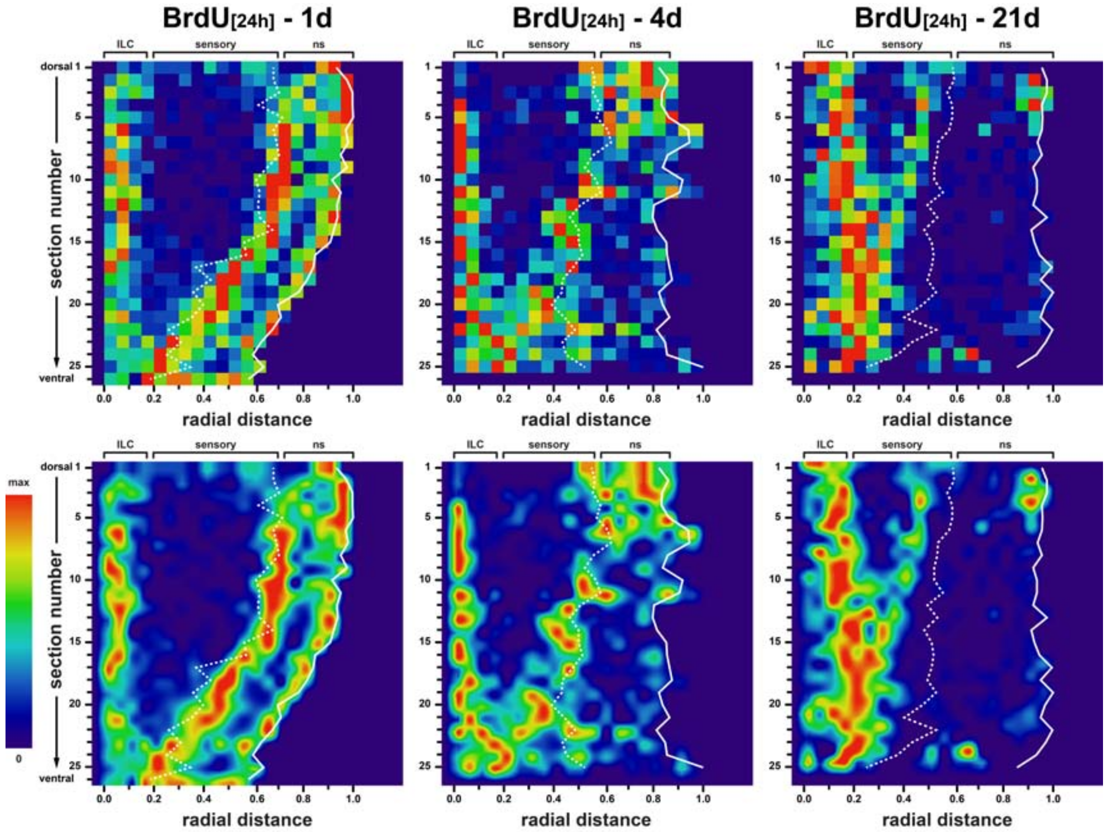

Supplementary Figure 2: Reconstruction of individual olfactory lamellae for different time points following 24h of continuous incubation with BrdU.

Heatmap plots denoting the frequency distribution of BrdU-positive cells on reconstructed surface views of individual olfactory lamellae at 1d (left), 4d (center), and 21d (right) following a 24h incubation with BrdU. The series at the top represents raw data, while images in the bottom panel have been processed by a bicubic scaling function for illustration purposes. The positions of cells were normalized to the largest radial extent of each OE and frequency distributions for each of 25 (4d, 21d) to 26 (1d) consecutive dorsal to ventral (top to bottom) sections were normalized to the maximum number of cells for the radial bins of each section.

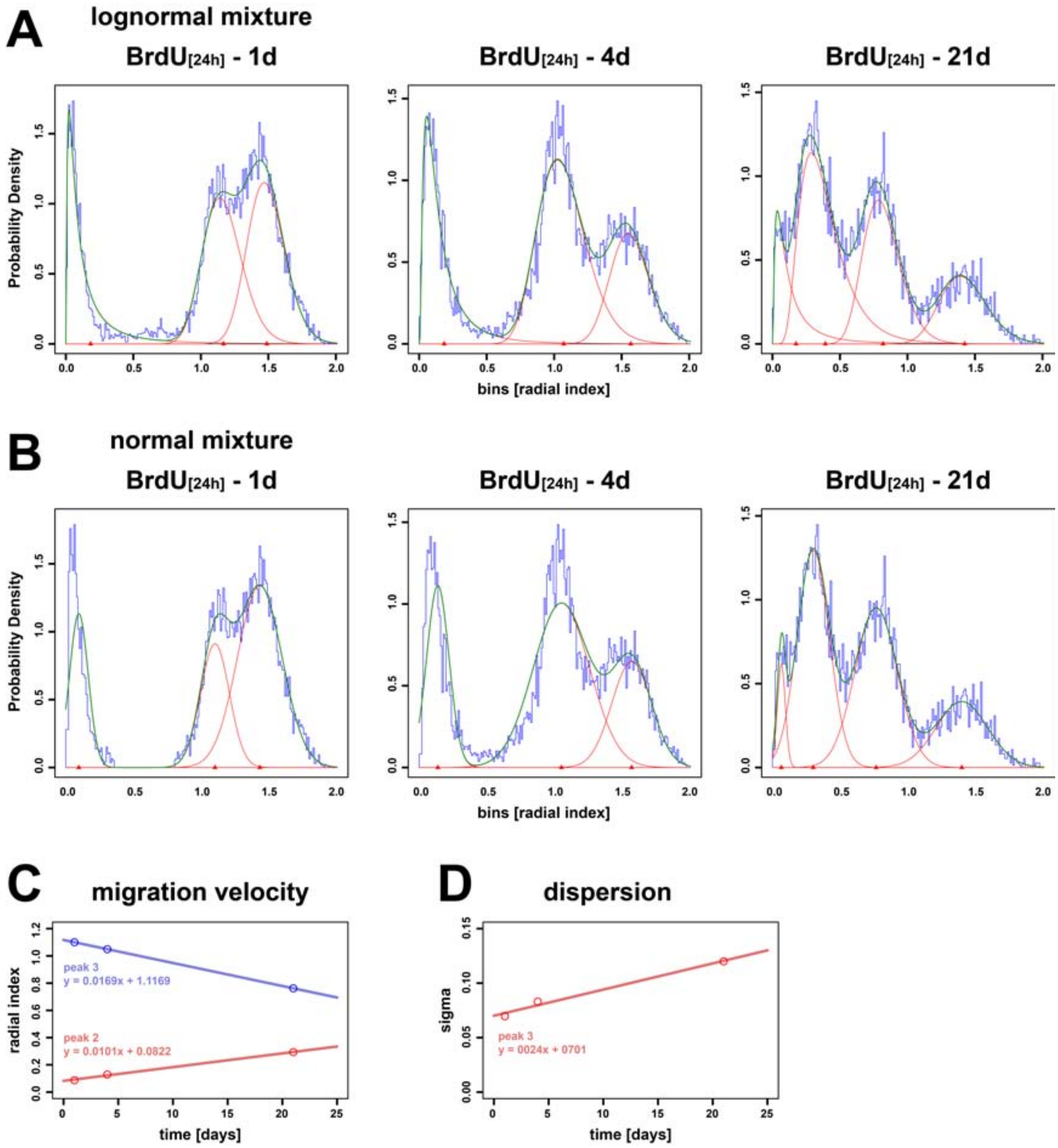

Supplementary Figure 3: Gaussian mixture models of timed BrdU distribution profiles.

Finite mixture model analysis, output of the mixdist* package in R** to fit individual lognormal components to combined BrdU profiles for 1d (left), 4d (center), and 21d (right) following a 24h BrdU incubation. The frequency distribution of the combined 14.365 data points for 1d experiment could be fitted to 3 lognormal distributions with the following parameters: peak 1: ρ = 0.2279, μ = 0.1833, σ=0.3145; peak 2: ρ = 0.3740, μ = 1.1677, σ = 0.1461; peak 3: ρ = 0.3981, μ = 1.4885, σ = 0.1395. The 10.957 data points for the 4d BrdU time point could be fitted to 3 lognormal distributions with following parameters: peak 1: ρ = 0.2603, μ = 0.1852, σ = 0.2042; peak 2: ρ = 0.4954, μ = 1.0703, σ = 0.1824; peak 3: ρ = 0.2443, μ = 1.5674, σ = 0.1464. The 7.463 data points for the 21d BrdU data could be fitted to 4 lognormal distributions with following parameters: peak 1: ρ = 0.1243, μ = 0.1746, σ = 0.2391; peak 2: ρ = 0.4190, μ = 0.3914, σ = 0.1855; peak 3: ρ = 0.2910, μ = 0.8189, σ = 0.1405; peak 4: ρ = 0.1738, μ = 1.4234, σ=0.1755. Data for each time point were obtained from 3 fish, 10 dorsal sections each.

* Peter Macdonald and with contributions from Juan Du (2012). mixdist: Finite mixture Distribution Models. R package version 0.5-4. http://CRAN.R-project.org/package=mixdist

** R Core Team (2015). R: A language and environment for statistical computing. R Foundation for Statistical Computing, Vienna, Austria. URL http://www.R-project.org/.

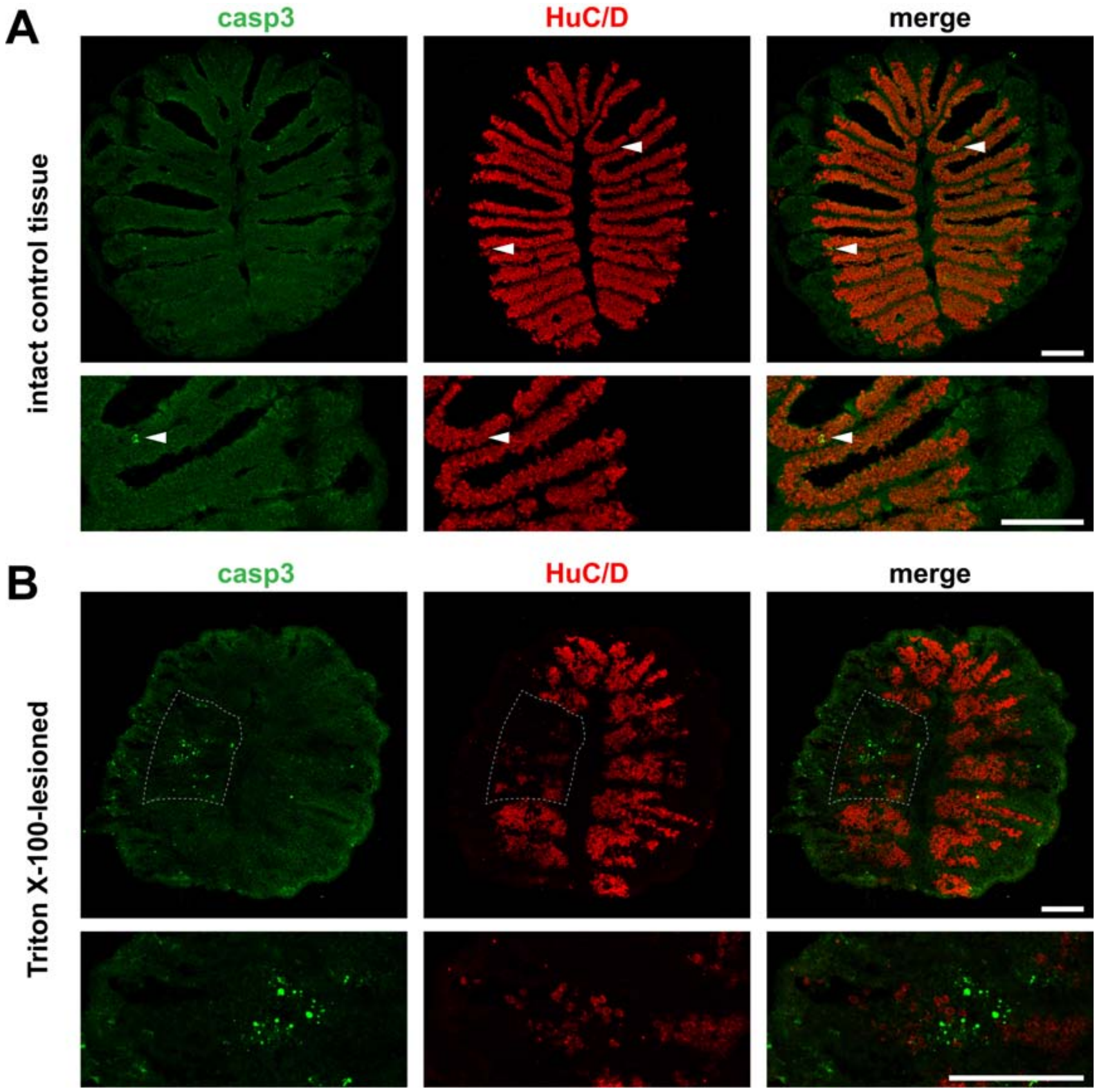

Supplementary Figure 4: Apoptotic cell death in the adult zebrafish olfactory epithelium.

A. Immunohistochemistry against the apoptotic marker cleaved caspase 3 (casp3, green, left) and the pan-neuronal marker HuC/D (red, centre) on full sections (top panel) and higher power views (bottom panel) of the intact zebrafish OE. Only occasional caspase 3-positive cells (arrowheads) can be detected. Scale bars: 100 µm.

B. Immunohistochemistry against the apoptotic marker cleaved caspase 3 (casp3, green, left and the pan-neuronal marker HuC/D (red, centre) on full sections (top panel) and higher power view (bottom panel) of the adult zebrafish OE 4 h after chemical lesion by nasal irrigation with 1% Triton X-100 for 90 s. Induction of caspase 3 activity is restricted to regions of the OE in which OSNs have been successfully damaged, indicated by the loss of HuC/D immunoreactivity (dotted lines). Scale bars: 100 µm.

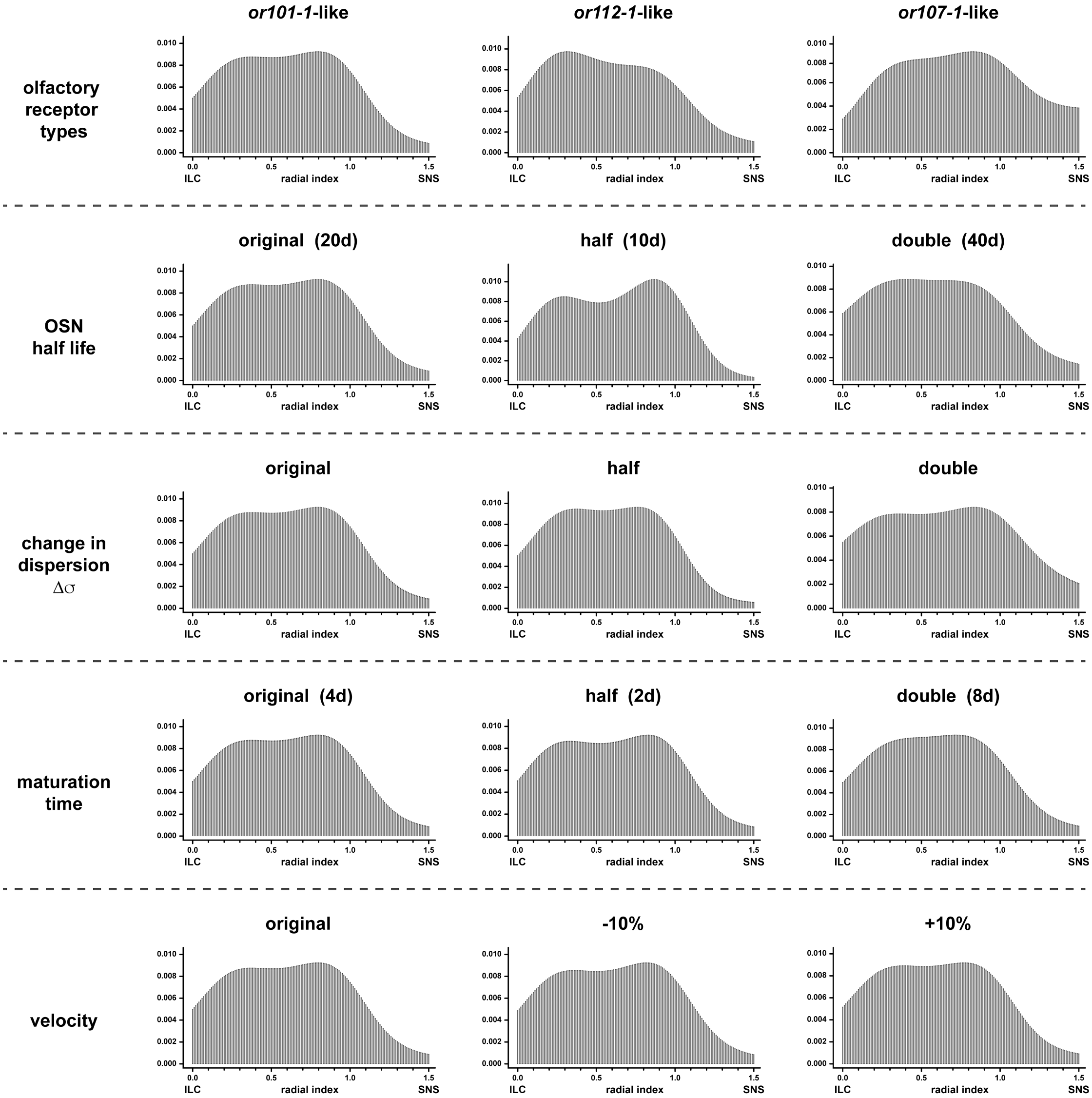

Supplementary Figure 5: Model output for variation in parameter settings.

The mathematical model derived from OSN neurogenesis generates OR-like distribution profiles based on simple parameters of cell dynamics, such as biased generation of OR-specific OSN subpopulations at the ILC and SNS, the half life of OSNs, their maturation time, the velocity of positional shifts across the OE, and changes in dispersion over time. The *or101-1*-like distribution (top, left) can be changed into an *or112-1*-like profile (top, centre) by adjusting the bias in generation of *or101-1*- and *or112-1*-expressing OSNs at the ILC and SNS from 0.64 (*or101-1*) to 1.21 (*or112-1*) but can be changed into an *or107-1*-like profile (top, right) by assuming increased speed of centrifugal shift as experimentally observed in Fig. 5B. Biased generation at the ILC and SNS and changes in OSN half life affect the model output more severely than changes in growth of dispersion, maturation time, or modest changes in velocity of transgression.

Supplementary Text 1. A mathematical model for OR expression profiles.

Instructions:

1. – open R* environment
2. – install the shiny** package and dependencies
3. – copy and paste the source code "app.R" below to the R console
4. – hit return to launch the browser
5. – parameters can be adjusted in the app browser to change the output in real time

* the R environment can be downloaded from: http://www.R-project.org/

** Winston Chang, Joe Cheng, JJ Allaire, Yihui Xie and Jonathan McPherson (2016). shiny: Web Application Framework for R. R package version 0.13.2. the package can be downloaded from: http://CRAN.R-project.org/package=shiny

app.R

```
library(shiny)

ui <- (fluidPage(
  plotOutput('plot'),
  hr(),
  h4("OR distribution model"),
  fluidRow(
    column(3,
         numericInput("x_input", "Maximum range", value = 1.5),
         numericInput("half_life", "Half life", value = 15),
         numericInput("maturation", "Time to maturation", value = 4),
         numericInput("proliferation_ILC", "proliferation at ILC", value = 1),
         numericInput("proliferation_SNS", "proliferation at SNS", value = 1)
    ),
    column(4,
          sliderInput("mean_ILC_init", "initital mean at ILC:",
                min = 0.0, max = 0.3, step = 0.01, value = 0.15),
          sliderInput("speed_ILC", "centrifugal migration speed:",
                min = 0.0, max = 0.05, step = 0.001, value = 0.015),
          sliderInput("sigma_ILC_init", "initial sigma at ILC:",
                min = 0.0, max = 0.3, step = 0.01, value = 0.15),
          sliderInput("sigma_ILC_spread", "dispersion of sigma from ILC:",
                min = 0.0, max = 0.02, step = 0.001, value = 0.008)
    ),
    column(4,
          sliderInput("mean_SNS_init", "initital mean at SNS:",
                min = 0.8, max = 1.5, step = 0.01, value = 1.0),
          sliderInput("speed_SNS", "centripetal migration speed:",
                min = 0.0, max = 0.05, step = 0.001, value = 0.015),
          sliderInput("sigma_SNS_init", "initial sigma at SNS:",
                min = 0.0, max = 0.3, step = 0.01, value = 0.15),
          sliderInput("sigma_SNS_spread", "dispersion of sigma from SNS:",
                min = 0.0, max = 0.02, step = 0.001, value = 0.008)
    )
  )
))
```

```
server <- shinyServer(function(input, output) {
      output$plot <- renderPlot({
        x <- seq(0, input$x_input, 0.01)
        time <- seq(1, 100, 1)
        survival_t <- 0.5^((time-1)/input$half_life)
        mat_step <- 1/input$maturation
        mat_t <- seq(mat_step, 1, mat_step)
        mat_diff <- length(time)-length(mat_t)
        maturation_t <- c(mat_t, rep(1, mat_diff))
        mean_ILC_end <- input$mean_ILC_init+((length(time)-1)*input$speed_ILC)
        mean_ILC_t <- seq(input$mean_ILC_init, mean_ILC_end, input$speed_ILC)
        sigma_ILC_t <- seq(from = input$sigma_ILC_init, by = input$sigma_ILC_spread,
 length.out=100)
        mean_SNS_end <- input$mean_SNS_init-((length(time)-1)*input$speed_SNS)
        mean_SNS_t <- seq(input$mean_SNS_init, mean_SNS_end, -input$speed_SNS)
        sigma_SNS_t <- seq(from = input$sigma_SNS_init, by = input$sigma_SNS_spread,
 length.out=100)
ILC_profiles <- function(mean_ILC_t, sigma_ILC_t) dnorm(x, mean_ILC_t, sigma_ILC_t,
 FALSE)
        ILC_profiles_t <- mapply(ILC_profiles, mean_ILC_t, sigma_ILC_t)
        ILC_profiles_t_norm <- t(t(ILC_profiles_t)/rowSums(t(ILC_profiles_t)))
        ILC_profiles_t_norm_m <- mapply("*",as.data.frame(ILC_profiles_t_norm), maturation_t)
        ILC_profiles_t_norm_m_s <- mapply("*",as.data.frame(ILC_profiles_t_norm_m),
 survival_t)
        ILC_profiles_t_norm_m_s_r <-
 ILC_profiles_t_norm_m_s*(input$proliferation_ILC/(input$proliferation_ILC+input$proliferatio
 n_SNS))
        ILC_t <- ILC_profiles_t_norm_m_s_r
SNS_profiles <- function(mean_SNS_t, sigma_SNS_t) dnorm(x, mean_SNS_t, sigma_SNS_t,
 FALSE)
        SNS_profiles_t <- mapply(SNS_profiles, mean_SNS_t, sigma_SNS_t)
        SNS_profiles_t_norm <- t(t(SNS_profiles_t)/rowSums(t(SNS_profiles_t)))
        SNS_profiles_t_norm_m <- mapply("*",as.data.frame(SNS_profiles_t_norm),
 maturation_t)
        SNS_profiles_t_norm_m_s <- mapply("*",as.data.frame(SNS_profiles_t_norm_m),
 survival_t)
        SNS_profiles_t_norm_m_s_r <-
 SNS_profiles_t_norm_m_s*(input$proliferation_SNS/(input$proliferation_ILC+input$prolifera
 tion_SNS))
        SNS_t <- SNS_profiles_t_norm_m_s_r
combined <- rowSums(SNS_t)+rowSums(ILC_t)
        combined <- combined/sum(combined)

        matplot(x, combined, type="h", col="black", lty=1, lwd=4, xlim=c(0, input$x_input),
 ylim=c(0, max(combined)), xlab="radial index", ylab="frequency", lend=1)
    })
})

shinyApp(ui = ui, server = server)
```
